# Identification of Cancer Related Genes Using a Comprehensive Map of Human Gene Expression

**DOI:** 10.1371/journal.pone.0157484

**Published:** 2016-06-20

**Authors:** Aurora Torrente, Margus Lukk, Vincent Xue, Helen Parkinson, Johan Rung, Alvis Brazma

**Affiliations:** 1 Instituto Gregorio Millán, Universidad Carlos III de Madrid, Leganés, Madrid, Spain; 2 European Molecular Biology Laboratory, European Bioinformatics Institute (EMBL-EBI), Hinxton, United Kingdom; 3 Cancer Research UK Cambridge Institute, University of Cambridge, Cambridge, United Kingdom; 4 Massachusetts Institute of Technology, Cambridge, Massachusetts, United States of America; 5 Department of Immunology, Genetics and Pathology, Clinical Sequencing Facility, Science for Life Laboratory, Uppsala, Sweden; University of Turin, ITALY

## Abstract

Rapid accumulation and availability of gene expression datasets in public repositories have enabled large-scale meta-analyses of combined data. The richness of cross-experiment data has provided new biological insights, including identification of new cancer genes. In this study, we compiled a human gene expression dataset from ∼40,000 publicly available Affymetrix HG-U133Plus2 arrays. After strict quality control and data normalisation the data was quantified in an expression matrix of ∼20,000 genes and ∼28,000 samples. To enable different ways of sample grouping, existing annotations where subjected to systematic ontology assisted categorisation and manual curation. Groups like normal tissues, neoplasmic tissues, cell lines, homoeotic cells and incompletely differentiated cells were created. Unsupervised analysis of the data confirmed global structure of expression consistent with earlier analysis but with more details revealed due to increased resolution. A suitable mixed-effects linear model was used to further investigate gene expression in solid tissue tumours, and to compare these with the respective healthy solid tissues. The analysis identified 1,285 genes with systematic expression change in cancer. The list is significantly enriched with known cancer genes from large, public, peer-reviewed databases, whereas the remaining ones are proposed as new cancer gene candidates. The compiled dataset is publicly available in the ArrayExpress Archive. It contains the most diverse collection of biological samples, making it the largest systematically annotated gene expression dataset of its kind in the public domain.

## Introduction

Discovery and cataloguing of gene expression in normal and disease conditions has been facilitated by generation of large gene expression datasets. Many of these sets have been deposited in public databases such as GEO [1] or ArrayExpress [2]. Combining different expression datasets has enabled studies of expression for tissues and cell types outside single experiment, has made possible addressing questions different to those posed in the original studies and has led to new biological insights that otherwise could not have been obtained [3]. One of the first examples of combination of large collections of human gene expression data was reported in [4]. The authors conducted an analysis of gene co-expression from ∼4,000 human microarray samples, followed by the assessment of the functional relevance and reproducibility of the detected patterns. Later, [5] constructed a database of ∼10,000 samples from normal and pathological human tissues, using five different Affymetrix platforms, with the aim to provide an integrated view of expression variance across hundreds of different tissue and disease types. Before these studies, most human microarray datasets, with exceptions such as [6], were commonly focusing on experiments comparing expression from only a small number of different samples. Also, [7] built a global gene expression map by integrating data from ∼5,000 human samples from a single microarray platform (Affymetrix HG-U133A). To span the full variety of expression, samples from many different cell and tissue types, disease states and cell lines, all data available at the time, were included. Six differentiated, major ‘continents’ of expression were revealed by the data: cell lines, hematopoietic system, incompletely differentiated tissues, brain, muscle and other solid tissues. More recently, [8] collected a heterogeneous dataset of ∼20,000 gene expression profiles from a variety of human samples and experimental conditions. The authors inferred a consensus transcriptional gene network based on mutual information and demonstrated its use to elucidate the function of disease genes. Moving to more recent microarray platforms (Affymetrix HG-U133 Plus 2.0), [9] set up a database of ∼3,000 gene expression samples from various tissues and disease states to associate expression patterns to phenotypes, in order to select phenotypically meaningful gene signatures. One of the largest datasets ever reported contained ∼78,000 samples from three species (human, mouse and rat) and four Affymetrix platforms, each representing different tissue types, disease states and cellular contexts [10]. The focus of the study was to investigate the extent of global gene dosage sensitivity, to infer the likely biological function of candidate genes on the basis of gene co-regulation and to identify recurrently disrupted human genes in genomically unstable cancers. More specifically focused on cancer, [11] performed an integrative analysis of five genome-wide platforms and one proteomic platform on ∼3,500 specimens from 12 cancer types to provide information for prediction of clinical outcomes. Most recently, [12] presented PRECOG, a pan-cancer resource of expression signatures that integrated gene expression from a diverse set of microarray platforms and RNA-seq. The data was collected from ∼18,000 human tumours across 39 malignancies for identification of predictors of overall patient survival.

The identification of genes associated with the development and progression of cancer and other genetic diseases has been the central goal of hundreds of microarray data projects. Most studies have addressed the analysis of differentially expressed genes, often involving one cancer type and/or several cancer subtypes. However, the sets of differentially expressed genes yielded by different studies of the same cancer have been reported to show little overlap [13, 14]. Rather than comparing the results of individual studies, it may thus be a better strategy to identify (larger and more robust) sets of genes by first integrating individual studies. An illustration of this scheme is given in [15], where the authors fitted a probeset-level mixed-effects linear model to a dataset which combined data from two different chip platforms, to identify genes differentially expressed in oral tongue squamous cell carcinomas. The result was compared to the one obtained for each study analysed separately. A substantial increase of the statistical power was observed in the meta-analysis scenario.

Mixed-effects linear models have been frequently used in microarray data studies [16–21]. These statistical models contain fixed effects for which inferences cannot be generalised to levels outside the design. Further, random effects they include are drawn from a population with a certain probability distribution. Compared to models considering only fixed effects, the inclusion of random effects allows a more precise estimation of sources of variability. This is particularly crucial in the detection of differentially expressed genes [22], for instance in case of cancer.

This work builds on the vast amount of raw microarray data available in ArrayExpress [2], containing both the data originally submitted to ArrayExpress as well as the data submitted to GEO [1] and automatically mirrored by ArrayExpress. We focused on a single array platform, the Affymetrix HG-U133Plus2 chip, integrating data from all experimental conditions, varying disease states, independent tissue types and different laboratories. All of the data, more than 40,000 samples were collected, pre-processed, filtered for quality control and curated, and produced a single dataset of ∼28,000 samples. This collection includes normal and diseased hematopoietic and solid tissues and cell lines, and provides a new, updated illustrative global map of human gene expression.

Unsupervised analyses of the dataset, such as dimension reduction by principal component analysis (PCA) and hierarchical clustering, yield results largely consistent with those obtained in [7], but provide a finer detail of the components discovered in the data. We then focused on a subset of samples consisting of solid tumours and the respective normal tissues with the aim to identify, with high statistical power, candidate genes that are involved in cancer. We propose a probeset-level mixed-effects linear model for the observed gene expression, which accounts for the variability due to several effects, such as the effect of the tissue of origin and the experiment where the sample was recorded. The latter partially reflects the laboratory-related effects which are known to be strong [23].

The main benefit of our model is that it allows evaluation of the significance of the (fixed) disease status effect. We use the corresponding corrected p-values to designate the probesets of a significant effect as candidates related to cancer processes, regardless of the tissue type. Applying the model to a subset of 5,938 solid tissue samples, we identified 1,285 potential cancer related genes. Gene set enrichment analysis reveals significant pathways deeply connected to the progression of cancer. The comparison of this set of genes to two human curated sets of known cancer genes shows significant overlaps (135 and 210, respectively). We propose the remaining genes in our list to be potentially novel cancer genes.

## Materials and Methods

### Gene expression dataset

Raw Affymetrix HG-U133Plus2 platform expression data was identified and downloaded from ArrayExpress data archive [2]. The initial dataset consisted of 40,871 CEL files from 1,768 experiments and included samples from a variety of normal and non-healthy cell and tissue types, as well as from cell lines. Replicated data files were identified by comparison of file sizes and MD5 check sums and removed. The remaining chips were tested for the quality using the Bioconductor package simpleaffy (see S1 Appendix for details). The number of samples taken forward to pre-processing and normalisation by frozen Robust Multiarray Average (fRMA) [24] was 30,899. After further quality control, based on the pre-processed data, the collection was frozen to 27,887 arrays and combined into a final gene expression data matrix.

For annotation consistency, original sample annotations were automatically re-annotated using Experimental Factor Ontology (EFO) [25]. EFO is a purpose-built ontology for systematic description of experimental variables used in high-throughput functional genomics datasets. The ontology builds on and combines parts of several other biological ontologies, including ontologies for anatomy, disease and chemical compounds. The content of ArrayExpress sdrf and idf files capturing the sample meta-data were scanned for all EFO terms followed by manual curation. The number of unique biological groups identified was 2,969. Of these, more than 1,000 contained only one sample, including single observations of more than 800 distinct cell lines (S1 Table).

### Principal Components Analysis

Principal Components Analysis was run on the final gene expression data matrix with probesets as variables and samples as statistical units. Similarly to and for comparison with [7], we focused on the first three principal components. For better handling of the size of the data matrix, we used Lapack C library compiled in 64-bit mode and distributed the computation using hadoop. Hadoop allows distributed processing of large datasets across clusters of computers using local computation and storage, helping to circumvent restricted memory problems of other standard software.

### Clustering

For further exploratory analysis, we first considered the clustering of biological groups, i.e., the sets of samples labelled with the same annotation. We reduced the dimension of the data, from samples to biological groups, in the following way. First, for each pair of samples we computed the correlation between their expression levels across the probesets. Next, for each pair of biological groups *G*_*m*_ and *G*_*n*_, we assessed their similarity by the average of the correlations between each sample from the first group and each sample from the second group:

$$similarity\left(G_{m},G_{n}\right)=\sum_{s_{m_{i}}\in G_{m}}\sum_{s_{n_{j}}\in G_{n}}\frac{cor(s_{m_{i}},s_{n_{j}})}{|G_{m}\left|\;\right|G_{n}|}.$$ (1)

Additionally, we considered the following dimension-reduced expression matrix *Y* = (*Y*_*mn*_) for the biclustering of probesets versus biological groups. For each probeset *p*_*m*_ and each biological group *G*_*n*_, we computed the average expression level of *p*_*m*_ across all samples in *G*_*n*_:

$$Y_{mn}={\bar{y}}_{n(p_{m})}=\sum_{s_{l}\in G_{n}}\frac{y_{ml}}{|G_{n}|},$$ (2)

where *y*_*ml*_ represents the expression level of probeset *p*_*m*_ in sample *s*_*l*_ ∈ *G*_*n*_. The corresponding heatmaps were computed using the R package gplots.

### Gene enrichment analysis

Gene over/under representation was performed by Gene Trail [26]; p-values were adjusted with the Benjamini-Hochberg method, and results were deemed significant at *p* < 0.05.

### Fitting a mixed-effects linear model

To assess the differences in a collection of *G* biological groups, we considered the between-group variance (BGV) for each particular probeset *p*:

$$BGV\left(p\right)=\sum_{n=1}^{G}\frac{N_{n}\;{\left({\bar{y}}_{n(p)}-{\bar{y}}_{\left(p\right)}\right)}^{2}}{G-1},$$

where *N*_*n*_ (*n* = 1, …, *G*) is the group size, ${\bar{y}}_{n(p)}$ is the mean expression level of probeset *p* across samples in the *n*-th group, and ${\bar{y}}_{\left(p\right)}$ is the probeset grand (overall) mean. It is well known that for most genes, the variability of gene expression across samples is small. Nevertheless, in many cases we found that even with little global variability, slight differences between sample groups are detectable. For example, with respect to tissue of origin as shown further in Results.

This suggests that even for genes whose expression level is almost constant across most tissue types, there might be some tissues, not all necessarily represented in our dataset, for which there is a statistically significant change in their expression level. Furthermore, if we focus on tumour versus normal tissues, and if a single tissue type is considered, small changes across the two disease statuses can be neglected when compared to genes whose change is larger in magnitude (see, for example [15]). In studies with the aim of finding reliable and well distinguishing marker genes, these subtle but firm changes are brushed on the side and commonly labelled as results significant statistically but not necessarily biologically.

As our dataset benefits from higher statistical power, we set to identify genes whose expression level changes depend on the disease status across many tissues of origin. If the change exists across different tissues, it will be detected even if the number of samples for some tissues would be too limited to be uncovered when analysed separately.

To that end, we propose to evaluate the effect of the disease status in the expression level of each probeset while accounting for the tissue type effect, using the following probeset-level mixed-effects linear model (i.e., incorporating both fixed and random effects):

$$y_{ijk}=\delta_{i}+t_{j}+t_{ij}+e_{k}+e_{ik}+\epsilon_{ijk}\;.$$ (3)

The response variable *y*_*ijk*_ represents here the expression level of the given probeset and is modelled as the addition of the fixed effect of the *i*-th *disease status* *δ*_*i*_, plus the partially-crossed random-effects factors *tissue type*, *t*_*j*_, and *experiment* in which it was obtained, *e*_*k*_, plus the interactions between the disease and the tissue type, *t*_*ij*_, and between the disease and the experiment, *e*_*ik*_. The term *ϵ*_*ijk*_ is the random error. The assumptions of the model are that the random variables *t*_*j*_, *e*_*k*_ and *ϵ*_*ijk*_ are independent and normally distributed. Note that the fixed effect term can be recoded as the average expression level of the gene, *μ*, plus the difference between cancer and normal tissues effect, *β*, which is our parameter of interest in this study.

For unbalanced designs (with different sizes across the different groups, as is our case), calculating a p-value for testing the null hypothesis *H*_0_: *β* = 0 versus the alternative *H*_1_: *β* ≠ 0 is not straight forward, as the distribution of the desired test statistic under *H*_0_ is not known. Therefore, for each probeset we tested the significance of the fixed-effects term by computing the ratio of its estimate $\hat{\beta }$ to the corresponding standard error, and considering an approximated *t*-distribution for this ratio. The degrees of freedom used in our computations are calculated as an upper bound given by the number of observations minus the number of fixed-effects parameters. This number leads to p-values that are anti-conservative for small samples [27] but to overcome this issue we considered classes with at least 20 replicates. Note that this is a general model that can be used on datasets with paired (normal vs cancer) samples of even larger dimensions.

To fit model (3) to each probeset, we used the R package lme4. The approximated p-values were corrected for multiple comparisons using the Benjamini-Hochberg method, which controls the false discovery rate (FDR). In addition, a restrictive test size of 0.01 was chosen. Further evaluation was carried out by performing a permutation test, where the empirical distribution was constructed by randomly permuting the disease status labels within each tissue type *B* = 10,000 times. Permutation p-values were calculated as fraction of permutation values that were at least as extreme as the original statistic. P-values were adjusted according to Benjamini-Hochberg to control for FDR and compared to those derived from non-permuted data. The same process allowed estimation of FDR using a standard permutation plug-in estimator. We used p-value cut point 0.01. *F* was the estimated expected number of false positives. We counted the number of significant tests in each permutation and computed *F* as the average of the number of probesets called significant from all *B* permutations. *S* denoted the number of probesets called significant using the correct labelling of the dataset. *p*_0_ was the estimated proportion of probesets that are truly null, and was computed as described in [28], with *λ* = 0.5. Then, the FDR was estimated as

$$\hat{FDR}=\frac{p_{0}\;F}{S}.$$

## Results

After collection, quality control, cautious ontology annotation and normalization we obtained a gene expression matrix of 54,675 probesets (mapping to 23,437 genes) and 27,887 samples, consistently annotated by EFO [25]. Unsupervised analysis of the matrix revealed a structure compatible with earlier observations on a smaller dataset of around 15,000 genes and 5,000 samples [7]. However, the larger amount of probesets and variety of samples allows higher resolution, such as identification of smaller subgroups within some regions of the map. Also, tissue types not included in previous dataset can be properly located.

### PCA to identify major ‘continents’

**Fig 1:**
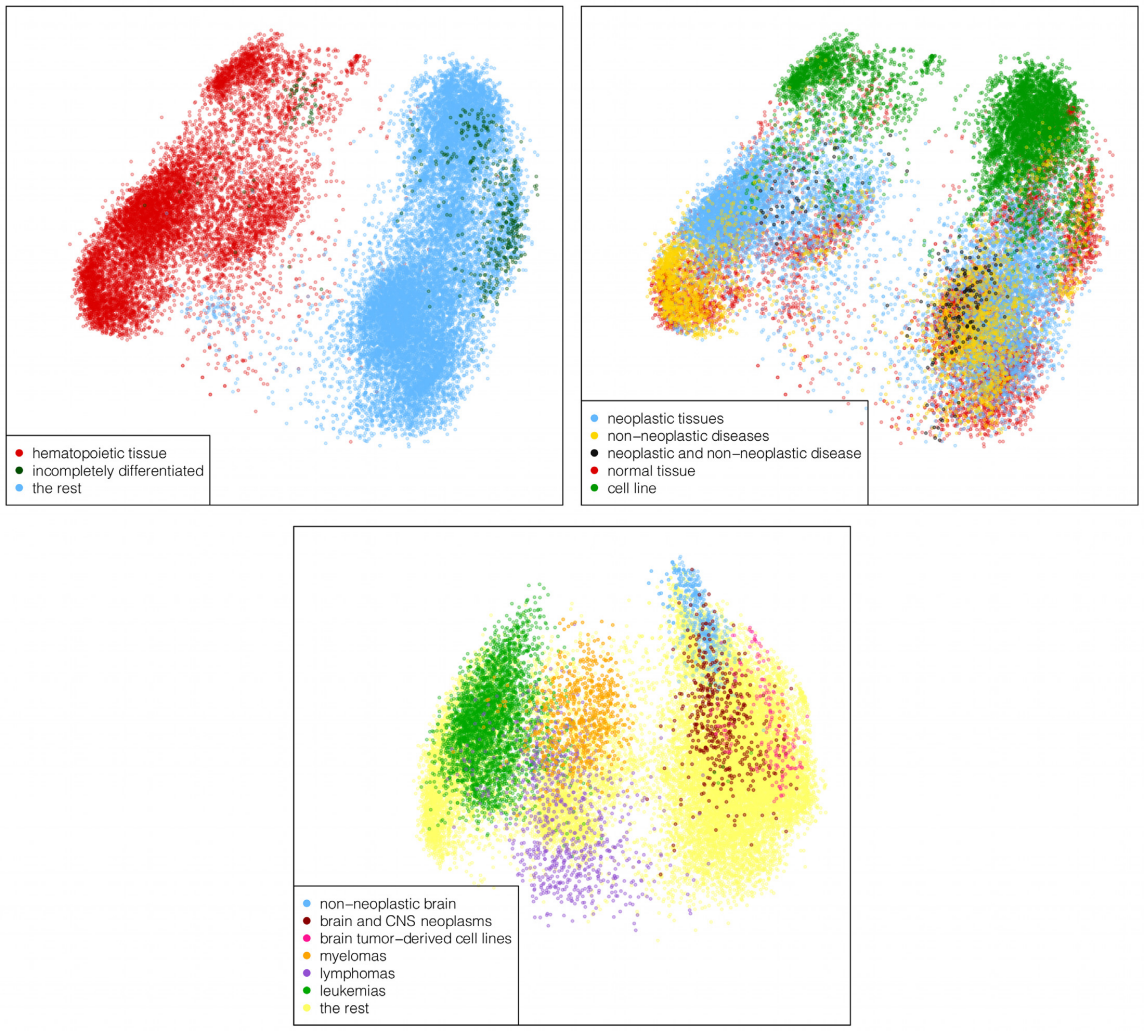
Principal components. On top, the first two principal components. The panel on the left illustrates the clear separation along the X-axis of the hematopoietic and non-hematopoietic material containing samples from both tissues and cell lines. The non-solid region includes blood, bone marrow, lymph nodes, tonsil, osteoclasts, spleen, sputum, thymus gland, bronchoalveolar lavage cells and derived cell lines. An elongated cluster including incompletely differentiated cells is also found. In the right panel, the Y-axis separates cell lines (top), neoplasias (middle) and non-neoplastic diseases (bottom), whereas normal tissues overlap with all three. The panel in the bottom shows the data for the first (X-axis) and third components (Y-axis). The hematopoietic axis (X-axis) allows detaching leukaemias from other blood neoplasias. Cell lines derived from brain tumours can be distinguished from their tissues of origin along this axis as well. The Y-axis detaches non-neoplastic central nervous system samples from tumoral ones, and separates myelomas from lymphomas.

An inspection of the PCA plots (see Fig 1) reveals that the first principal component clearly bisects non-solid and solid tissues. The segregation occurs also among cell lines which otherwise tend to cluster among each other, as opposed to clustering close to the tissue of origin. An elongated cluster of induced pluripotent stem cells, mesenchymal stem cells, progenitor cells and other incompletely differentiated tissues emerges within the solid section. In the non-solid region, also hematopoietic precursors and plasmablast cells cluster together. This axis also separates leukaemias from myelomas and lymphomas (see the bottom panel of Fig 1). This result is a major improvement over the lower resolution global map reported by [7] where only leukaemic and non-leukaemic tissues were displayed. Moreover, the map presented here includes an experiment of bone samples (annotated as “bone;trans-iliacal bone;menopausia;”) located closer to the non-solid area than to the solid one, suggesting high levels of hematopoietic material present in the sample. The first principal component accounts for a 16.72% of the total variance in the dataset.

The second principal component, accounting for 9.57% of the variance, locates cell lines and non-neoplastic diseases at opposite extremes of the plot, with neoplasias spreading between both. Normal tissues are distributed all along the axis. The third principal component (4.97% of the variance) detaches healthy brain and spinal cord from central nervous system malignancies in the solid cluster, and lymphomas from myelomas in the non-solid cluster.

Note that these results coincide with those obtained in [7], but provide a finer detail. We can thus identify the first principal component with their hematopoietic axis, the second one with their malignancy axis, and the third one with their neurological axis.

### Clustering identifies finer details in the map

#### Hematopoietic tissues, cell lines and solid tissues

**Fig 2:**
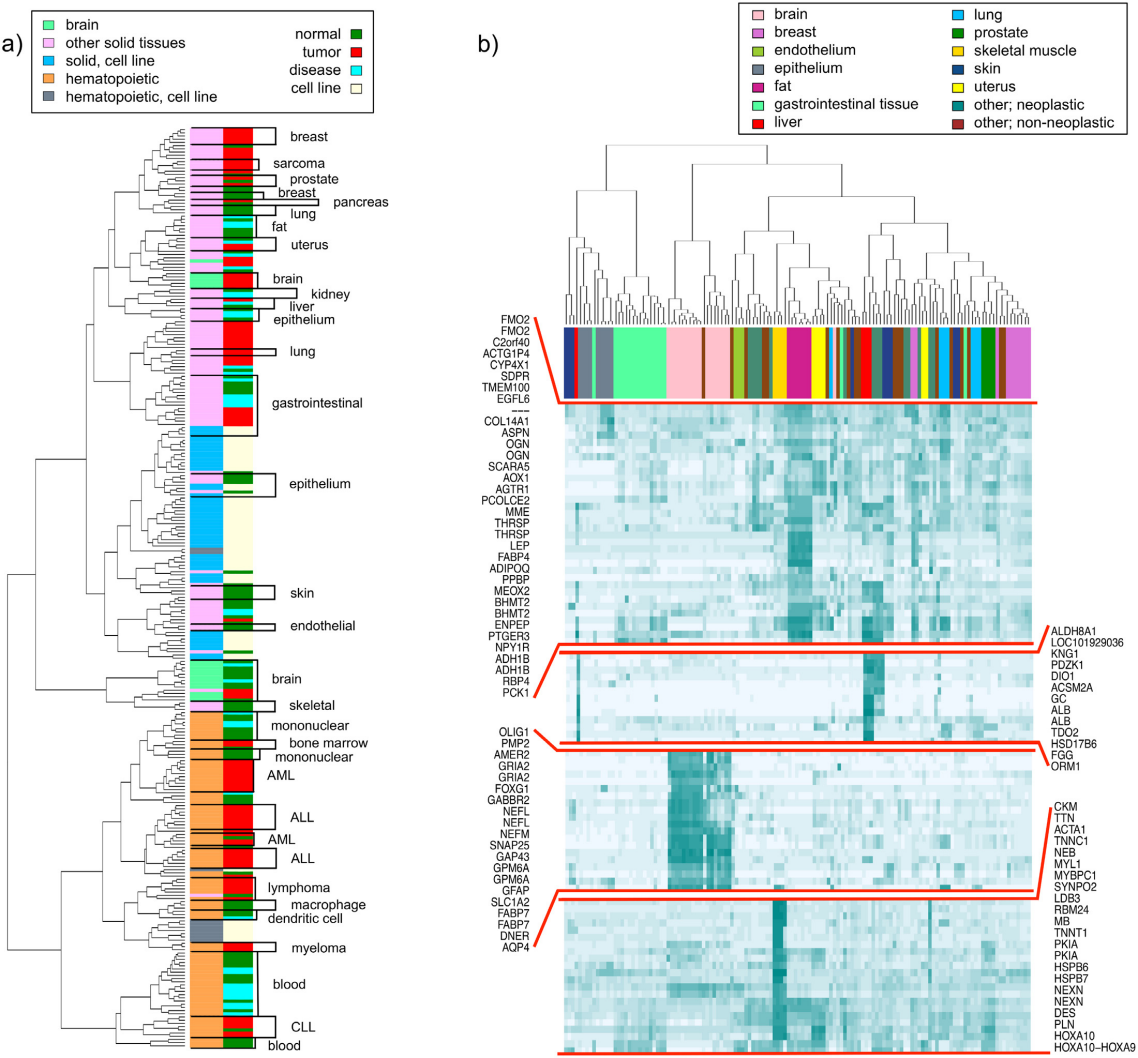
Clustering of biological groups with at least 20 observations. a) Clustering of biological groups using average correlations. Only the 20,000 most variable probesets are accounted for. The groups are recoded by colours to display the largest clusters in the dendrogram. The colour labels on the right hand side specify groups of healthy, cancerous or diseased tissues or cell lines. Illustrative groups of tissues are also highlighted. b) Clustering of biological groups of solid tissues against the 1,000 most variable probesets. Many clusters, identified by visual inspection, include genes overexpressed in one or more tissues of origin. From top to bottom, clusters C.8, C14, C.18 and C.29 display probesets with high activity in adipose tissue, liver, brain and skeletal muscle and heart, respectively.

For the cluster analysis, in order to guarantee statistical significance, we only considered the 287 biological groups with at least 20 samples. The filtering decreased the number of samples to 18,106, of which 2,830 were cell lines, 7,013 were hematopoietic tissues, and 8,263 were solid tissues. Similarity measure Eq (1) used for clustering of the biological groups was in the range of (0.6317, 0.9953). The resulting heatmap is found in S1 Fig. To allow a better separation among groups, as reflected in the wider ranges of measure Eq (1), we additionally reduced the dimension of the biological groups by selecting the *n* most variable probesets, with *n* = 20,000, 10,000, 5,000, 1,000 and 500 (see S2, S3, S4, S5 and S6 Figs). Despite progressive reduction of the number of probesets the hierarchical structure between biological groups remained the same. As shown in Fig 2a), the hierarchical tree clearly distinguishes between solid and non-solid samples, correspondent to the two largest clusters, as expected from the PCA, and contains an outstanding solid cluster formed by non-neoplastic brain. In the non-solid region, hematopoietic-derived cell lines appear together, as expected. We find smaller clusters of different neoplastic diseases: lymphomas, myelomas and leukaemias, separating AML, CLL and ALL. Also, dendritic cells, macrophages, and non-leukaemic peripheral blood form clear groups in the tree. Again, the biological group annotated as “bone;trans-iliacal bone;menopausia;”, which contains samples from a single experiment, emerges on the hematopoietic side. On the non-hematopoietic side, solid cell lines also tend to cluster together, irrespectively of their tissue of origin and similarly to the results obtained in [7]. There are also small clusters of specific tumours (brain, colon and soft tissues) and a broad group of diverse tumours. Within the non-neoplastic solid tissues, we identify several groups such as adipose tissue, endothelial cells, airway epithelial cells or gastrointestinal tissues. Interestingly, neoplastic and non-neoplastic gastrointestinal tissues are positioned together in the dendrogram to form a larger group.

#### Non-cell lines, non-hematopoietic tissues

Given the distinct behaviour of cell lines and known separation of solid and hematopoietic samples, we next focused on the non-hematopoietic samples. After discarding 59 and 96 biological groups comprising, respectively, all cell lines and hematopoietic tissues, the dataset was reduced to 8,263 samples from 132 biological groups of at least 20 replicates.

A separate hierarchical analysis of the solid tissues, analogous to that of the previous section, led to the heatmaps in S7, S8, S9, S10, S11 and S12 Figs, where we considered all or a reduced set of probesets. By visual inspection, several of the observed clusters are in agreement with earlier results in [7]. Notably, a collection of gastrointestinal tissues forms a large and compact group, that is, irrespective of the disease status. Similarly, tight groups are observed for samples of prostate, myometrium and liver. Although brain samples cluster together, the cluster is further subdivided to neoplastic and non-neoplastic branches. On the contrary, breast cancer and normal breast are markedly separated, as well as cancerous and non-cancerous lung, skin or pancreas, the latter examplifying a tissue not included in [7]. Other apparent solid regions include endothelial cells, airway epithelial cells, adipose tissue, sarcomas, incompletely differentiated cells, kidney, skeletal muscle and endometrium.

In summary, the benefit of integration of vast amounts of tissue types is best demonstrated in the analysis of clustering results. Compared to previous results in [7], greater numbers of samples have a strong effect for the resolution of ‘continents’ and ‘continental’ features of the human expression map. Separation of different types of leukaemias is revealed. Some tissues, such as liver or colon, have respective expressions similar to the associated tumours. In contrast, other cancers, such as lung or brain, have the expression patterns of tumours and corresponding normal samples clearly separated.

#### Clustering genes across non-cell lines, non-hematopoietic tissues

In addition to the previous analysis, we constructed a heatmap for the 1,000 most variable probesets and plotted these against the biological groups using a reduced expression matrix computed as described in Eq 2 (see S13 Fig). The leaves in the dendrogram on top correspond to the biological groups. The branch ordering was fixed to be the same as in S11 Fig. The labels in the right margin include the genes the probesets are mapping to, identified at the NetAffx Analysis Center [29] provided by Affymetrix. As expected, probesets targeting the same gene tend to cluster together. By visual inspection, we identified 44 clusters, many of which include genes that are overexpressed in one or more tissues of origin. Gene enrichment, calculated with Gene Trail [26], discloses a large agreement between gene profiles and Kyoto Encyclopedia of Genes and Genomes (KEGG) and Gene Ontology (GO) categories. Fig 2b) includes some illustrative examples. In particular, Cluster C.18, where probesets show a higher activity in brain samples, has an overrepresentation of genes related to ‘nervous system development’, or ‘neuron differentiation’. Genes in cluster C.8, whose activity is increased in adipose tissues, are involved in categories such as ‘PPAR signaling pathway’ or ‘lipid metabolic process’. Genes exhibiting a high activity in liver (cluster C.14) have overrepresented functions such as ‘hemostasis’, ‘vitamin transport’ or ‘cellular amino acid and derivative metabolic process’. Also, probesets highly expressed in skeletal muscle and heart samples (cluster C.29), have significance in categories such as ‘muscle structure development’ or ‘heart contraction’.

### Identification of cancer genes using mixed-effects linear model

#### Paired cancer/normal data to identify significant cancer-related effects

To discover genes with largest expression difference between normal and corresponding tumorigenic tissues, which may not be detectable in smaller datasets due to lower statistical power, we selected tissues with at least 20 samples in both normal and corresponding non-metastatic tumour tissues. This resulted in 15 pairs of biological groups, spanning 5,938 samples (Table 1).

**Table 1:** Group sizes in paired tissues.

Tissue type	Cancer	Normal
airways and lung	309	447
bone	37	23
brain	330	474
breast	926	222
colon	575	168
fat	72	448
gastro-intestinal	293	79
head and neck	96	84
kidney	44	269
mesenchymal	129	54
pancreas	73	48
prostate	88	21
skin	61	148
smooth muscle	73	123
uterus	79	145

Sizes of the biological groups with at least 20 replicates for which both normal and cancer samples are available.

**Fig 3:**
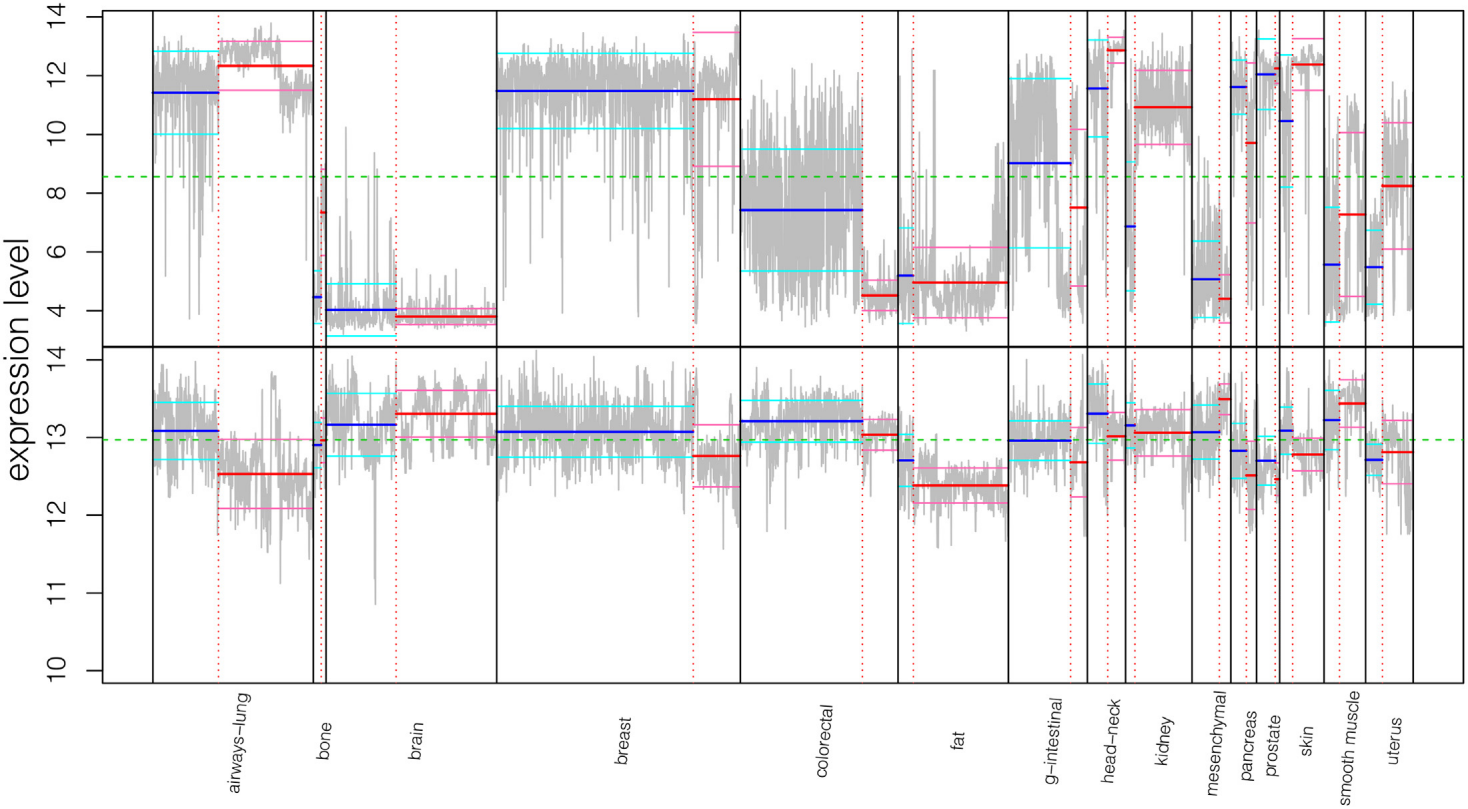
Extreme expression level profiles. Expression levels of the most variable probeset (202286_s_at), on top, and a low-variability probeset corresponding to housekeeping gene GAPDH (217398_x_at), at the bottom, across all tissues for which there are at least 20 replicates of untreated, normal and cancerous samples. Samples from the same tissue of origin are displayed together, grouped by disease status. The green dashed line represents the overall mean; blue and red solid lines show the mean of cancerous and normal groups, respectively, whereas cyan and pink solid lines describe their respective dispersion, given by the within group standard deviation.

Only 5,934 (10.85%) probesets have a drastically large BGV, i.e., are outliers (see S14 Fig). However, most of the remaining probesets exhibit a noticeable change in their expression level across some of the conditions. For illustration, we first show the expression levels of two probesets exhibiting characteristic, distinc behaviours, across the 5,938 samples, where samples are ordered to show correspondent cancerous and normal tissues consecutively (Fig 3). Probeset expression levels are depicted in gray; the grand mean ${\bar{y}}_{\left(p\right)}$ is represented by a green, dashed line, and the cancerous and normal group means ${\bar{y}}_{n(p)}$ by blue and red, solid lines, respectively. To visualise the variability within groups, we computed the group variances, $\sigma_{n(p)}^{2}$, and drew the lines corresponding to ${\bar{y}}_{n(p)}\pm \sigma_{n(p)}$, for cancerous and normal groups in cyan and pink, respectively. We first considered the probeset with largest BGV (202286_s_at), which according to Affymetrix annotations maps to gene TACSTD2. The gene is involved in biological processes such as ‘re-entry into mitotic cell cycle’ or ‘positive regulation of stem cell differentiation’. It is apparent (Fig 3, top panel) that the probeset has a different behaviour depending on the tissue type, and in some cases, depending on the disease status. We then selected probeset 217398_x_at, annotated as well known housekeeping gene GAPDH, to represent probesets with low variability. Evidently, the gene has noticeable expression differences between the groups, either due to the disease status or to the tissue type (Fig 3, bottom panel). This is not entirely surprising as some housekeeping genes are known to exhibit alterations in levels of expression even in normal tissues, let alone in tumorigenesis.

**Fig 4:**
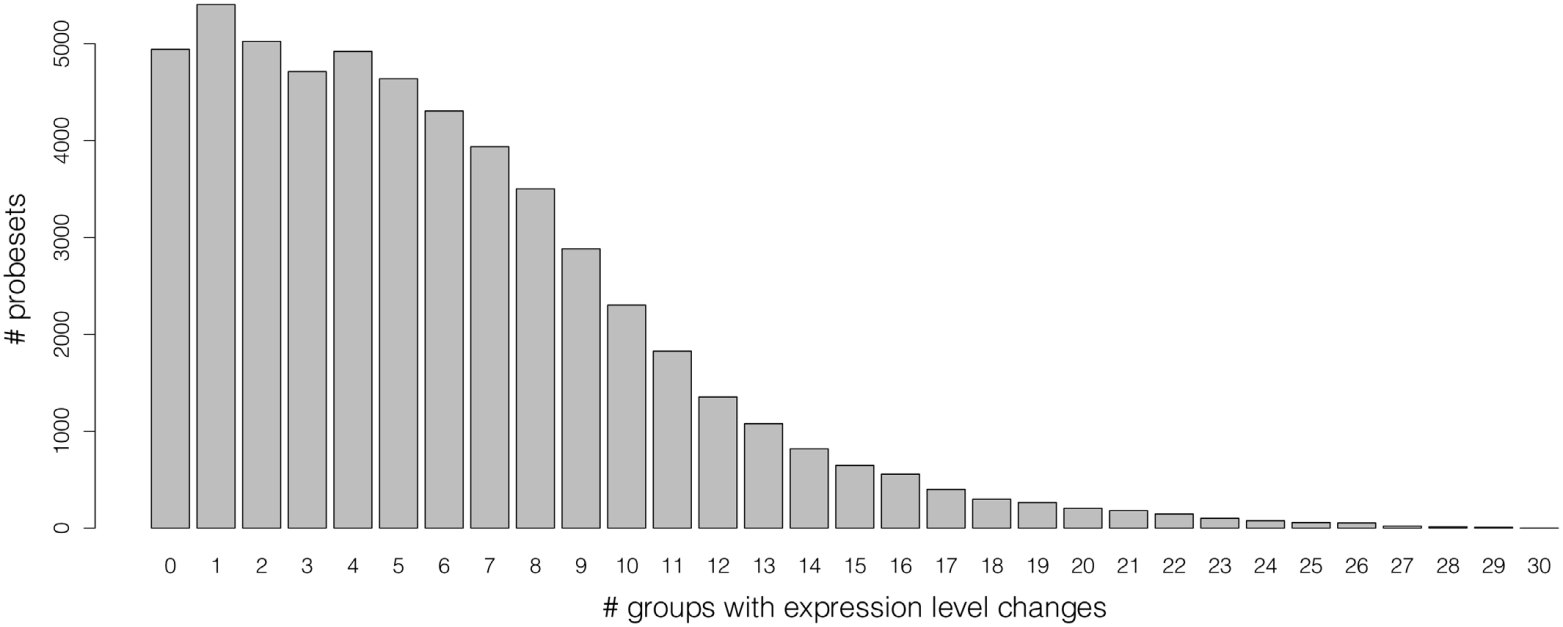
Expression level changes across tissue types and disease status. Distribution of the number of groups for which there is a clear change in the expression level of the probeset. The values are quantified by having either the group mean minus the group standard deviation above the overall mean or the group mean plus the group standard deviation below the overall mean.

Between these two diametric cases, with respect to the tissue of origin or to the disease status, it is possible to detect evident expression changes of most of the probesets. More precisely, we computed for each probeset the number of groups for which either ${\bar{y}}_{n(p)}-\sigma_{n(p)}>{\bar{y}}_{\left(p\right)}$ or ${\bar{y}}_{n(p)}+\sigma_{n(p)}<{\bar{y}}_{\left(p\right)}$. The distribution of these counts is displayed on Fig 4. Only 4,942 probesets out of the 54,675 (9.04%) do not show such variability across any of the 30 conditions.

Thus, we fitted model (3) with 2 disease statuses, 15 tissue types and 212 experiments, and considered an approximated *t* distribution with degrees of freedom equal to 5,936 for the ratio of the parameter of interest $\hat{\beta }$ to its standard error. After multiple comparison correction, 1,835 probesets were identified with significant effect of the disease status (p-values smaller than 0.01). The results were further validated by a permutation test (see the QQ-plot in S15a Fig). The empirical p-values are smaller than those obtained from the model fit except for values in the tails. However, there is a large agreement between both tests as the number of probesets found significant with the permutation procedure at level 0.01 was 2,542, with an overlap of 1,743 probesets of the original list (∼94.99%). Also, the FDR estimation from the permutation scheme supports the validity of our list of significant probesets. The average number of significant probesets after random relabelling and multiple comparison correction (false positive estimate) was 0.0019. Thus the FDR estimate was 1.035422 × 10^−6^. The QQ-plot in S15b Fig compares adjusted p-values from the original data to those obtained after one random permutation of the disease status labels where none of the probesets were found to be significant.

Significant probesets were mapped to the corresponding genes by NetAffx Analysis Center [29] (S2 Table). Of these, 137 probesets did not match any gene. The remaining mapped to 1,285 unique genes and 97 had multiple matchings. The 1,835 probesets were ranked according to their p-values and, for illustration purposes, the top 100 for which the effect of the disease was most significant were dissected further. The p-values for the top 100 were less than 3.632501 × 10^−6^. Six of them were not mapped to any gene, whereas the remaining 94 corresponded to one of 84 unique genes or 7 multiple matching genes (Table 2).

**Table 2:** Genes mapping to the top 100 probesets.

AATF (*)	AK056098	ANAPC7 (**)	**ANXA11** (**)
ARNTL (**)	**ASPN** (**)	**BLM** (*)	C1orf21 (**)
***CASK*** (**)	CBFA2T2 (**)	CBX2 (**)	CD80 (**)
CDCA4 (**)	CHP1 (**)	CHTOP (**)	CPSF1^[1]^
CSE1L (*)	**CXCL10** (*)	CYB5D2	DCAF13 (**)
**DDX11** (**)	DLEU2^[2]^	DNMT1 (*)	DUSP5P1 (**)
**EMR2**	FAM122B (**)	FANCA (*)	FCGR1A^[3]^
FCGR1B (**)	FDX1 (**)	FLJ41455	GABBR1^[4]^
GINS4 (**)	GMPS (*)	HAUS3 (**)	HAUS8 (**)
HCG18	HELLS (*)	**LEF1** (**)	*LOC100506639*^[5]^
LPCAT1 (**)	**MAP3K12** (**)	MATR3^[6]^	MIR1204^[7]^
MMP24-AS1	**MMP9** (*)	MTBP (**)	NCOA6 (**)
NCOR1 (**)	NR2C1 (**)	NR2C2AP (**)	NR3C2 (*)
NSMCE2 (**)	NSUN2 (**)	**NUDT1** (**)	PARP9 (**)
PDS5A (**)	POLQ (**)	PPP2CB (**)	PRDM13 (**)
**PTP4A3** (**)	RBL1 (**)	RFX5	**RGS1** (**)
RGS16 (**)	RNPS1 (**)	RP11-353N14.2	RP11-932O9.10
RPS15A (**)	**RPS6KB1** (**)	S100PBP (*)	SALL4 (**)
SMOC2 (**)	SNORA72 (**)	STX12 (**)	**TDO2** (**)
THOC2 (**)	TIAL1 (**)	TMEM194A (**)	TMEM246 (**)
**TREM2** (**)	U2SURP (**)	USP32 (*)	VPS13D
VWA1 (**)	ZDHHC2 (**)	ZHX1-C8orf76	ZNF174 (**)
ZNF680	ZNF692 (**)	ZNF7 (**)	

^[1]^ CPSF1 (**) /// MIR1234 (**) /// MIR6849 /// MIR939 (**)

^[2]^ DLEU2 (**) /// MIR15A (**)

^[3]^ FCGR1A (**) /// FCGR1B (**) /// FCGR1C

^[4]^ GABBR1 (**) /// UBD (*)

^[5]^ LOC100506639 /// ZNF131 (**)

^[6]^ MATR3 (**) /// SNHG4 (**)

^[7]^ MIR1204 /// PVT1 (*)

List of genes mapped to by the top 100 probesets, proposed as candidates to be connected to cancer processes, irrespectively of the tissue type. Probesets 216677_at, 229948_at, 235229_at, 235363_at, 241569_at and 243379_at, are not mapped to any gene. Entries mapped by more than one probeset are displayed in italic; multiple matchings are shown with a superindex. One, two and no asterisks correspond to genes which have been found in the Atlas of Genetics and Cytogenetics in Oncology and Haematology database [33] to be related, possibly related or not related to cancer processes, respectively. Genes in bold-face have been identified in [34] as overexpressed in cancer. Gene CASK and multiple matching LOC100506639 /// ZNF131, in italic, are mapped to by two and three probesets, respectively. Additionally, genes (in multiple matchings) PVT1 and UBD and CPSF1, MIR1234, MIR939, DLEU2, MIR15A, FCGR1A, FCGR1B, GABBR1, ZNF131, MATR3 and SNHG4 are identified to be related and possibly related to cancer, respectively.

Additionally, the effect sizes of the selected probesets are identified on a volcano plot of disease effect versus negative log10-transformed p-values (see S16 Fig). The overall disease effect sizes appear modest, even for probesets with considerably small p-values. This is not unexpected, as the power of the dataset is substantial and allows identifying significant effects of small size. Also, it supports the idea that most of the genes associated to cancer will have a small overall, tissue-independent, disease effect, while the interaction between disease and tissue type plays an ultimate role in expression levels.

#### Gene enrichment analysis

Gene over/under representation for this list of candidate probesets reveals many significant KEGG and GO pathways categories related to cancer. In particular, KEGG categories are: ‘Cell cycle’, ‘DNA replication’, ‘Olfactory transduction’, ‘Spliceosome’, ‘Homologous recombination’, ‘Pyrimidine metabolism’, ‘Oocyte meiosis’, ‘Base excision repair’ and ‘Mismatch repair’, whereas GO categories include ‘DNA repair’, ‘cell proliferation’, ‘G-protein coupled receptor protein signaling pathway’ or ‘positive regulation of mitotic cell cycle’.

An illustrative example of the utility of our method is given by well-known genes BRCA1 and BRCA2, ranked 502-nd and 597-th, respectively. Though the relation of these genes to cancer has not been established on the basis of gene expression changes, our method identifies both of them. They appear in significant GO pathways such as ‘regulation of cell cycle’, ‘DNA repair’, ‘response to DNA damage stimulus’, ‘cell proliferation’ or ‘double-strand break repair via homologous recombination’, in which they are known to play essential roles. It is worth noting that the 63 genes predicted to be co-regulated with BRCA1 and BRCA2 by [10], overlap with 39 genes from our collection of candidate genes.

Interestingly, the significant KEGG and GO pathways categories corresponding to the top 100 probesets include ‘TGF-beta signaling pathway’, ‘Cell cycle’, ‘DNA repair’, ‘mitotic cell cycle’, ‘response to DNA damage stimulus’, ‘positive regulation of cell proliferation’ and ‘cell division’.

#### List validation

Next, we searched for independent evidence of a connection of these genes to cancerous processes. Several cancer databases (see for example [30–33]) include lists of genes that have been related to the disease, based on different aspects such as methylation in various cancer types, somatic mutations or evidences of tumour suppressing activity. There are also previous initiatives enumerating genes overexpressed in different cancer types compared to their normal tissue of origin; see e.g. [34, 35]. However, the overlap among these lists is small, and is so also with our panel of genes.

Our first benchmark for validating our list of cancer genes was against the Atlas of Genetics and Cytogenetics in Oncology and Haematology [33], a large peer-reviewed database based on genetics abnormalities and focused on protein coding and non-coding genes implicated in cancer. The Atlas provides a list of 1,432 cancer related genes, the largest of such collections to the best of our knowledge. For the sake of clarity, we will refer to the Atlas list as L1. After discarding multiple matchings, we observed 135 of 1,285 genes in our candidates in L1. Of these, 12 were among the top 84 unique genes of our top 100 probesets (Table 2, indicated with an asterisk).

To assess the statistical significance of the counts, we used a hypergeometric test, in which the ‘gene universe’ is the collection of 23,437 genes which, conforming to Affymetrix NetAffx Analysis Center, the 54,675 probesets present on the used array platform are mapping to. Note that the entire collection of genes includes 1,367 genes from L1. According to the test, the probability of randomly drawing a list of 1,285 (84) genes with an overlap with L1 equal to or larger than 135 (12) is 1.393924 × 10^−11^ (0.00339165).

Next, we studied the relation of each of these 135 genes with the cancer processes reported in the Atlas. For 9, such relation is simply cancer predisposition. Additional 22 genes are connected to only one type of cancer. The rest include genes connected to many types of cancer, such as the tumour suppressor genes AXIN1 (9 types), BRCA1 (7), BRCA2 (12), MMP11 (11) and SOCS1 (13), or the oncogenes CSE1L (9), DNMT3A(6), EZH2 (13), FGFR2 (7) and PTTG1 (7). There are also 4 genes connected to more than 15 different types of cancer (S3 Table). Moreover, a pathway analysis of these genes reveals 21 significant KEGG categories, 12 of which correspond to some type of cancer.

The Atlas also provides a second list of other 27,411 genes possibly related to cancer, but for which no evidence has yet been found. We refer to this second collection of genes as L2. Though this is not a validated list, we compared, for completeness, our list to the set of 13,174 genes from L2 included in the HG-U133Plus2. We found an overlap of 944 genes between lists, and an overlap of 60 for the top 84 (Table 2, indicated with double asterisks). The p-values corresponding to the hypergeometric test for these counts are 1.394372 × 10^−39^ and 0.002936396, respectively. The significant KEGG categories revealed in gene over/under representation for these 944 genes involve transcription, replication and repair pathways, such as ‘DNA replication’, ‘Spliceosome’, ‘Cell cycle’, ‘Homologous recombination’, ‘Mismatch repair’, ‘Base excision repair’ and ‘MAPK signaling pathway’, and also ‘Pathways in cancer’.

The significant pathways reported for the 206 genes that do not appear in either L1 or L2 include ‘Replisome’, ‘Replication fork’ and ‘DNA replication, synthesis of RNA primer’.

We then analysed the overlap of our candidate list with an assembly of genes overexpressed in cancer with respect to the tissue from which this originated. This second benchmark, referred here as collection L3, contains 2,929 probesets mapped to 2,397 genes, which are described as overexpressed in different types of cancer after pairwise comparison [34]. We found 210 unique genes (247 probesets) in common with L3, 16 of which are among the top 84 genes (Table 2, indicated in bold-face, more details available in S4 Table). The p-values for the hypergeometric test for the whole list and for the top 84 genes are 2.358516 × 10^−12^ and 0.01041105, respectively. The significant KEGG pathways include ‘Cell cycle’, ‘DNA replication’ and ‘Non-homologous end-joining’, related to replication and repair and cell growth and death.

It is worth mentioning that the intersection with L3 comprises 154 genes not included in L1, whereas 59 additional candidate genes are found in alternative databases [30–32, 35].

In summary, these results serve as validation for the proposed model (3) and support the hypothesis that our collection of genes may effectively include many novel cancer genes.

## Discussion

The identification of genes related to cancer processes is one of the central goals in genome-wide experiments; however, different studies on the same cancer type often lead to results with little overlap [13, 14]. Thus, the use of meta-analysis to integrate diverse datasets and to benefit from a higher statistical power has revealed as a better strategy.

In this work, we have combined data from hundreds of publicly available experiments that use the Affymetrix HG-U133Plus2 array, jointly comprising tens of thousands individual assays. The annotation of such dataset allows delineation of a human gene expression map which is in agreement with that in [7], but more diverse. In particular, the PCA of the full data and the hierarchical clustering of the biological groups with at least 20 replicates enable, for instance, to discriminate different types of leukaemia, or to observe that for some tissue types, expression levels of normal and cancer states are more similar while for others, the tissue of origin is less similar to the related tumour than to some other tissue. Also, focusing on the most variable probesets allows finding meaningful clusters of genes that are highly expressed in some tissue types and, correspondingly, have an overrepresentation in pathways related to these tissues.

To benefit from the statistical power of this large dataset, we have proposed a probeset-level mixed-effects linear model for the observed expression. Our model accounts for random effects such as the experiment and the tissue of origin, and also for a fixed effect, the one of interest, given by the disease status (normal vs cancer). The inclusion of the tissue type as a random factor allows our results to be generalised to tissue types other than the ones included in our dataset, whereas no inference can be done to other diseases. For paired groups, we retained only tissues with at least 20 untreated normal and 20 untreated tumour samples. The fitting of the model to the subset of paired data allowed the identification, after appropriate correction for multiple comparisons, of a collection of 1,835 probesets (mapping to 1,285 unique genes) for which the disease status carries small but significant effect.

Gene enrichment analyses revealed significant categories related to cell division, DNA repair or G-protein coupled receptor protein signaling pathway, deeply connected to the progression of cancer. This supports the hypothesis that our list may include novel cancer genes.

Further independent validation was obtained by means of external, large databases that provide lists of cancer related genes. Several oncogenes, tumour suppressor genes and cancer predisposition genes emerge from comparisons to these lists. Overlaps larger than expected by chance have been shown. Also, the gene enrichment analysis for this subset of genes revealed several tissue-specific cancer pathways. It is worth noting that our proposed list of genes, which was obtained exclusively by identification of differential expression, includes genes whose connection to cancer processes has been previously established by other means than gene expression, for instance, on the basis of somatic mutations (e.g. the genes BRCA1, BRCA2). The genes that are not present in the external databases are proposed as new candidate cancer genes. We expect that our findings will help provide further fundamental insights in the regulatory pathways involved in cancer processes and that future research will support them.

## Supporting Information

S1 Appendix Data pre-processing and quality control. Description of the pre-processing and quality control steps and parameters.
(PDF)

S1 Table Samples and Biological groups. Collection of 27,887 annotated samples retrieved from ArrayExpress along with the biological group; the original experiments and assay names are given in the format ‘Experiment_CELfile’.
(XLS)

S1 Fig Heatmap; all biological groups and all probesets. Heatmaps for the average pairwise correlations between samples from any two biological groups with at least 20 observations. All probesets are accounted for in the computation of the correlations. The range for the similarity measure is (0.6317, 0.9953). The colour labels display smaller clusters in the hierarchical tree.
(PDF)

S2 Fig Heatmap; all biological groups and 20,000 most variable probesets. Heatmap for the average pairwise correlations between samples from any two biological groups with at least 20 observations. Only the 20,000 most variable probesets are accounted for in the computation of the correlations. The range for the similarity measure is (0.3012, 0.9946). The colour labels display smaller clusters in the hierarchical tree.
(PDF)

S3 Fig Heatmap; all biological groups and 10,000 most variable probesets. Heatmap for the average pairwise correlations between samples from any two biological groups with at least 20 observations. Only the 10,000 most variable probesets are accounted for in the computation of the correlations. The range for the similarity measure is (0.1352, 0.9938). The colour labels display smaller clusters in the hierarchical tree.
(PDF)

S4 Fig Heatmap; all biological groups and 5,000 most variable probesets. Heatmap for the average pairwise correlations between samples from any two biological groups with at least 20 observations. Only the 5,000 most variable probesets are accounted for in the computation of the correlations. The range for the similarity measure is (−0.0288, 0.9940). The colour labels display smaller clusters in the hierarchical tree.
(PDF)

S5 Fig Heatmap; all biological groups and 1,000 most variable probesets. Heatmap for the average pairwise correlations between samples from any two biological groups with at least 20 observations. Only the 1,000 most variable probesets are accounted for in the computation of the correlations. The range for the similarity measure is (−0.3591, 0.9960). The colour labels display smaller clusters in the hierarchical tree.
(PDF)

S6 Fig Heatmap; all biological groups and 500 most variable probesets. Heatmap for the average pairwise correlations between samples from any two biological groups with at least 20 observations. Only the 500 most variable probesets are accounted for in the computation of the correlations. The range for the similarity measure is (−0.4359, 0.9965). The colour labels display smaller clusters in the hierarchical tree.
(PDF)

S7 Fig Heatmap; solid groups and all probesets. Heatmap for the average pairwise correlations between samples from any two solid groups with at least 20 observations, accounting for all the probesets in the computation of the correlations. The range for the similarity measure is (0.7164, 0.9920). The colour labels display smaller clusters in the hierarchical tree.
(PDF)

S8 Fig Heatmap; solid groups and 20,000 most variable probesets. Heatmap for the average pairwise correlations between samples from any two solid groups with at least 20 observations, accounting for the 20,000 most variable probesets in the computation of the correlations. The range for the similarity measure is (0.3869, 0.9907). The colour labels display smaller clusters in the hierarchical tree.
(PDF)

S9 Fig Heatmap; solid groups and 10,000 most variable probesets. Heatmap for the average pairwise correlations between samples from any two solid groups with at least 20 observations, accounting for the 10,000 most variable probesets in the computation of the correlations. The range for the similarity measure is (0.1704, 0.9896). The colour labels display smaller clusters in the hierarchical tree.
(PDF)

S10 Fig Heatmap; solid groups and 5,000 most variable probesets. Heatmap for the average pairwise correlations between samples from any two solid groups with at least 20 observations, accounting for the 5,000 most variable probesets in the computation of the correlations. The range for the similarity measure is (0.0247, 0.9893). The colour labels display smaller clusters in the hierarchical tree.
(PDF)

S11 Fig Heatmap; solid groups and 1,000 most variable probesets. Heatmap for the average pairwise correlations between samples from any two solid groups with at least 20 observations, accounting for the 1,000 most variable probesets in the computation of the correlations. The range for the similarity measure is (−0.1312, 0.9938). The colour labels display smaller clusters in the hierarchical tree.
(PDF)

S12 Fig Heatmap; solid groups and 500 most variable probesets. Heatmap for the average pairwise correlations between samples from any two solid groups with at least 20 observations, accounting for the 500 most variable probesets in the computation of the correlations. The range for the similarity measure is (−0.2424, 0.9955). The colour labels display smaller clusters in the hierarchical tree.
(PDF)

S13 Fig Heatmap; solid groups vs 1,000 most variable probesets. Heatmap for the expression level of the 1,000 most variable probesets averaged over the samples included in each biological group with at least 20 observations. The range for this similarity measure is (2.6984, 14.4581). The colour labels display the same clusters as those in S11 Fig. The probeset labels report the name of the genes they are mapping to. Probesets mapping to the same gene are clustered together.
(PDF)

S14 Fig BGV for all probesets across paired tissues. The BGV ranges from 0.051 to 2,047.575, but only 10.85% of the probesets show a BGV really high (greater than 128.1, the ‘maximum’ whisker).
(PDF)

S15 Fig **a) Permutation test QQ-plot.** Quantiles of the adjusted permutation and observed p-values in log_10_ scale. Except for very extreme results observed due to resolution of attainable p-values in the permutation test, the observed p-values are larger than those obtained with the permutation test. **b) QQ-plot of correct vs shuffled disease labels.** After random permutation of the disease labels within each tissue type and multiple testing correction, none of the probesets are called significant.
(PDF)

S16 Fig Disease effect volcano plot. Plot of the disease effect, irrespective of the tissue type, versus the negative log 10-transformed p-values.
(PDF)

S2 Table Significant probesets. The list of 1,835 significant probes, for which there is a significant effect of the disease status, along with the corrected p-values, and the genes or set of genes they are mapping to. They are ordered according to increasing p-values. There are 1,285 unique genes and 97 multiple matchings.
(TXT)

S3 Table Genes found in the Atlas. The 135 unique genes found in the list L1 from the Atlas of Genetics and Cytogenetics in Oncology and Haematology are alphabetically ordered and displayed in bold-face. The types of cancer they have been related to are also shown.
(XLS)

S4 Table Genes overexpressed in cancer. The 210 unique genes found in the list L3 identified in [34] are alphabetically ordered and displayed in bold-face. The types of cancer they have been related to are also shown.
(XLS)
