# Supplementary material for: Identification of Cancer Related Genes Using a Comprehensive Map of Human Gene Expression
Source: PLoS One. 2016 Jun 20;11(6):e0157484. doi: 10.1371/journal.pone.0157484 (PMC4913919; doi:10.1371/journal.pone.0157484)
Supplement: S1 Appendix — Description of the pre-processing and quality control steps and parameters. (PDF) [file pone.0157484.s001.pdf]

Data pre-processing and quality control

After collecting the data, we carried out the following anlayses. For a first round of quality control we used four single-array filters (implemented in the Bioconductor package `simpleaffy`); we considered that the given chip was of good quality if the corresponding values lay within the indicated range.

- scaling factor: $(-2, 2)$
- average background: $(5.2, 7.2)$
- percentage of present calls: > 35
- RNA degradation: < 4.5

Next, we preprocessed the raw data set using fRMA and batches of size 1. The resulting expression levels were used to compute the relative log expression (RLE) and the generalized normalized unscaled standard error (GNUSE), each of which provide a value for each probeset of each array; these values were used to run the second round of quality control of the data and thus discard low quality chips. For each array and each quality measure we had a distribution of (54.675) values, that can be summarized through the median and the interquartile range (IQR). The chips that were considered to be of low quality were those whose median (or IQR) was an outlier in the collection of 30.915 medians (or IQRs), where outliers are considered to be any values that lie more than one and a half the IQR below the first quartile (i.e., $< Q_1 - 1.5xIQR$) or above the third quartile ($> Q_3 + 1.5xIQR$).
Since the batches in the fRMA method were of size 1, no further normalization was needed.
