# Supplementary material for: Identification of Cancer Related Genes Using a Comprehensive Map of Human Gene Expression
Source: PLoS One. 2016 Jun 20;11(6):e0157484. doi: 10.1371/journal.pone.0157484 (PMC4913919; doi:10.1371/journal.pone.0157484)
Supplement: S3 Fig — Heatmap for the average pairwise correlations between samples from any two biological groups with at least 20 observations. Only the 10,000 most variable probesets are accounted for in the computation of the correlations. The range for the similarity measure is (0.1352, 0.9938). The colour labels display smaller clusters in the hierarchical tree. (PDF) [file pone.0157484.s005.pdf]

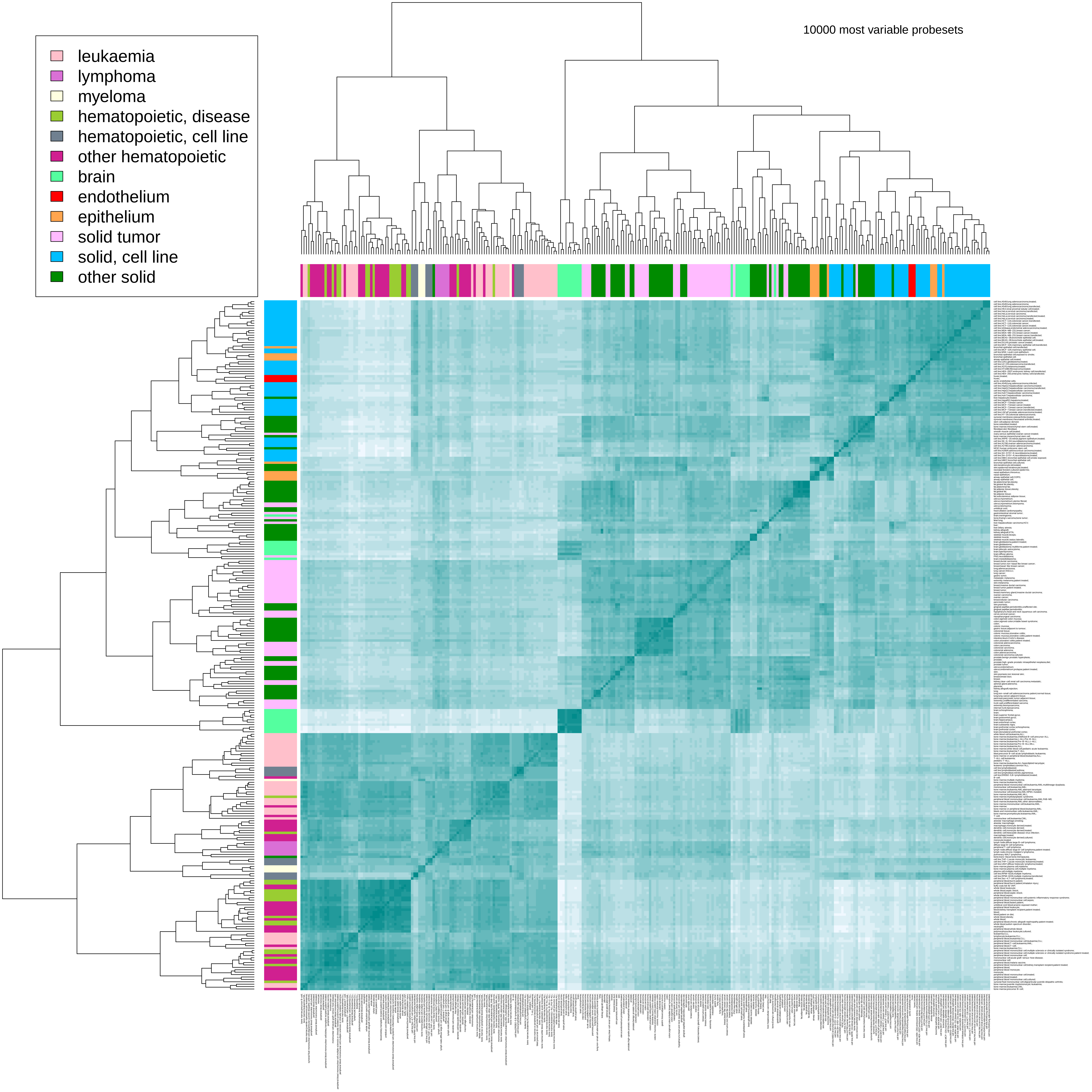
10000 most variable probesets
leukaemia
lymphoma
myeloma
hematopoietic, disease
hematopoietic, cell line
other hematopoietic
brain
endothelium
epithelium
solid tumor
solid, cell line
other solid
