# Supplementary material for: Identification of Cancer Related Genes Using a Comprehensive Map of Human Gene Expression
Source: PLoS One. 2016 Jun 20;11(6):e0157484. doi: 10.1371/journal.pone.0157484 (PMC4913919; doi:10.1371/journal.pone.0157484)
Supplement: S7 Fig — Heatmap for the average pairwise correlations between samples from any two solid groups with at least 20 observations, accounting for all the probesets in the computation of the correlations. The range for the similarity measure is (0.7164, 0.9920). The colour labels display smaller clusters in the hierarchical tree. (PDF) [file pone.0157484.s009.pdf]

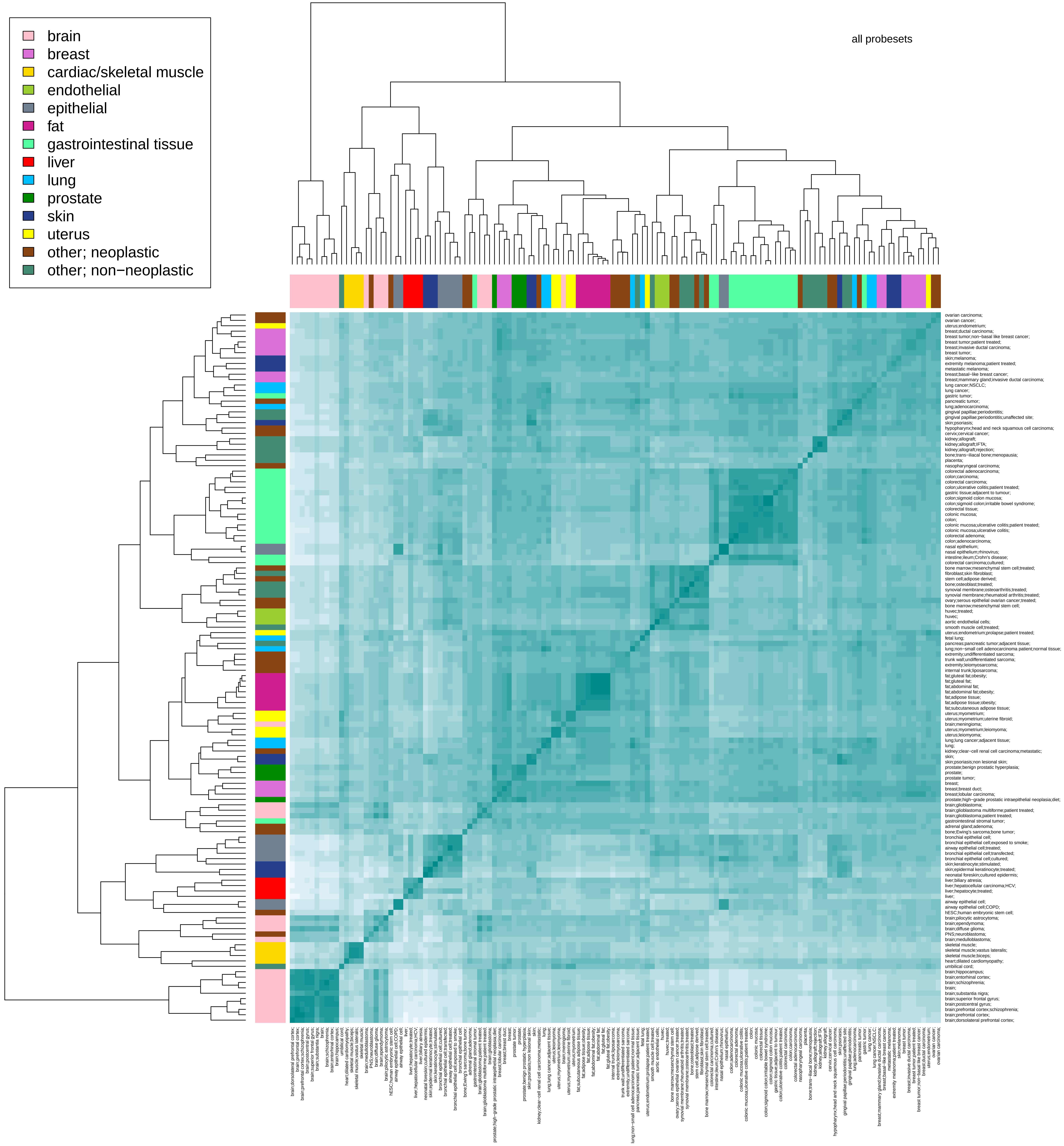

brain
breast
cardiac/skeletal muscle
endothelial
epithelial
fat
gastrointestinal tissue
liver
lung
prostate
skin
uterus
other; neoplastic
other; non−neoplastic
all probesets
ovarian carcinoma;
ovarian cancer;
uterus;endometrium;
breast;ductal carcinoma;
breast tumor;non−basal like breast cancer;
breast tumor;patient treated;
breast;invasive ductal carcinoma;
breast tumor;
skin;melanoma;
extremity melanoma;patient treated;
metastatic melanoma;
breast;basal−like breast cancer;
breast;mammary gland;invasive ductal carcinoma;
lung cancer;NSCLC;
lung cancer;
gastric tumor;
pancreatic tumor;
lung;adenocarcinoma;
gingival papillae;periodontitis;
gingival papillae;periodontitis;unaffected site;
skin;psoriasis;
hypopharynx;head and neck squamous cell carcinoma;
cervix;cervical cancer;
kidney;allograft;
kidney;allograft;IFTA;
kidney;allograft;rejection;
bone;trans−iliacal bone;menopausia;
placenta;
nasopharyngeal carcinoma;
colorectal adenocarcinoma;
colon;carcinoma;
colorectal carcinoma;
colon;ulcerative colitis;patient treated;
gastric tissue;adjacent to tumour;
colon;sigmoid colon mucosa;
colon;sigmoid colon;irritable bowel syndrome;
colorectal tissue;
colonic mucosa;
colon;
colonic mucosa;ulcerative colitis;patient treated;
colonic mucosa;ulcerative colitis;
colorectal adenoma;
colon;adenocarcinoma;
nasal epithelium;
nasal epithelium;rhinovirus;
intestine;ileum;Crohn's disease;
colorectal carcinoma;cultured;
bone marrow;mesenchymal stem cell;treated;
fibroblast;skin fibroblast;
stem cell;adipose derived;
bone;osteoblast;treated;
synovial membrane;osteoarthritis;treated;
synovial membrane;rheumatoid arthritis;treated;
ovary;serous epithelial ovarian cancer;treated;
bone marrow;mesenchymal stem cell;
huvec;treated;
huvec;
aortic endothelial cells;
smooth muscle cell;treated;
uterus;endometrium;prolapse;patient treated;
fetal lung;
pancreas;pancreatic tumor;adjacent tissue;
lung;non−small cell adenocarcinoma patient;normal tissue;
extremity;undifferentiated sarcoma;
trunk wall;undifferentiated sarcoma;
extremity;leiomyosarcoma;
internal trunk;liposarcoma;
fat;gluteal fat;obesity;
fat;gluteal fat;
fat;abdominal fat;
fat;abdominal fat;obesity;
fat;adipose tissue;
fat;adipose tissue;obesity;
fat;subcutaneous adipose tissue;
uterus;myometrium;
uterus;myometrium;uterine fibroid;
brain;meningioma;
uterus;myometrium;leiomyoma;
uterus;leiomyoma;
lung;lung cancer;adjacent tissue;
lung;
kidney;clear−cell renal cell carcinoma;metastatic;
skin;
skin;psoriasis;non lesional skin;
prostate;benign prostatic hyperplasia;
prostate;
prostate tumor;
breast;
breast;breast duct;
breast;lobular carcinoma;
prostate;high−grade prostatic intraepithelial neoplasia;diet;
brain;glioblastoma;
brain;glioblastoma multiforme;patient treated;
brain;glioblastoma;patient treated;
gastrointestinal stromal tumor;
adrenal gland;adenoma;
bone;Ewing's sarcoma;bone tumor;
bronchial epithelial cell;
bronchial epithelial cell;exposed to smoke;
airway epithelial cell;treated;
bronchial epithelial cell;transfected;
bronchial epithelial cell;cultured;
skin;keratinocyte;stimulated;
skin;epidermal keratinocyte;treated;
neonatal foreskin;cultured epidermis;
liver;biliary atresia;
liver;hepatocellular carcinoma;HCV;
liver;hepatocyte;treated;
liver;
airway epithelial cell;
airway epithelial cell;COPD;
hESC;human embryonic stem cell;
brain;pilocytic astrocytoma;
brain;ependymoma;
brain;diffuse glioma;
PNS;neuroblastoma;
brain;medulloblastoma;
skeletal muscle;
skeletal muscle;vastus lateralis;
skeletal muscle;biceps;
heart;dilated cardiomyopathy;
umbilical cord;
brain;hippocampus;
brain;entorhinal cortex;
brain;schizophrenia;
brain;
brain;substantia nigra;
brain;superior frontal gyrus;
brain;postcentral gyrus;
brain;prefrontal cortex;schizophrenia;
brain;prefrontal cortex;
brain;dorsolateral prefrontal cortex;
