# Supplementary figures and images for: Identification of Cancer Related Genes Using a Comprehensive Map of Human Gene Expression
Source: PLoS One. 2016 Jun 20;11(6):e0157484. doi: 10.1371/journal.pone.0157484 (PMC4913919; doi:10.1371/journal.pone.0157484)

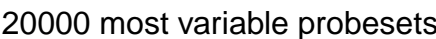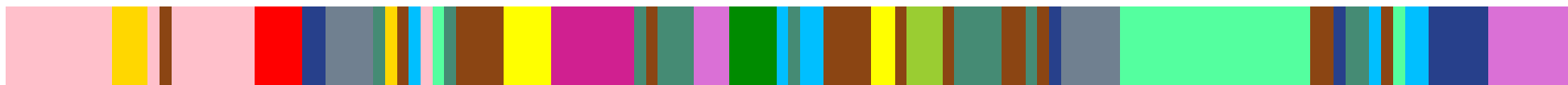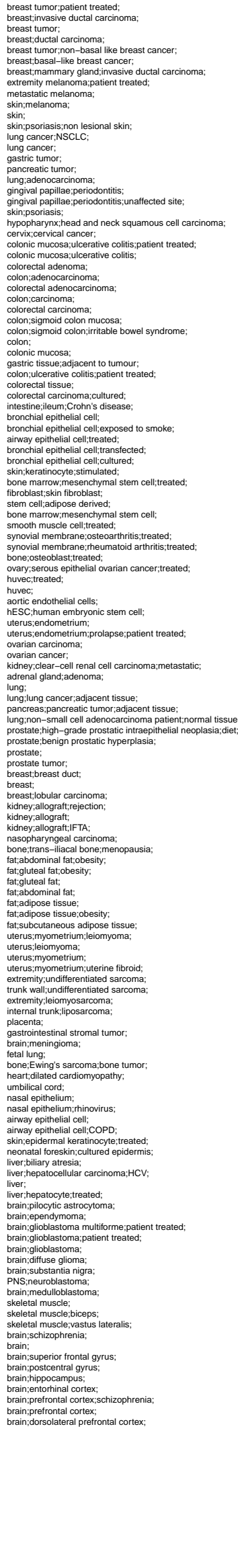

Supplement: S8 Fig — Heatmap for the average pairwise correlations between samples from any two solid groups with at least 20 observations, accounting for the 20,000 most variable probesets in the computation of the correlations. The range for the similarity measure is (0.3869, 0.9907). The colour labels display smaller clusters in the hierarchical tree. (PDF) [file pone.0157484.s010.pdf]

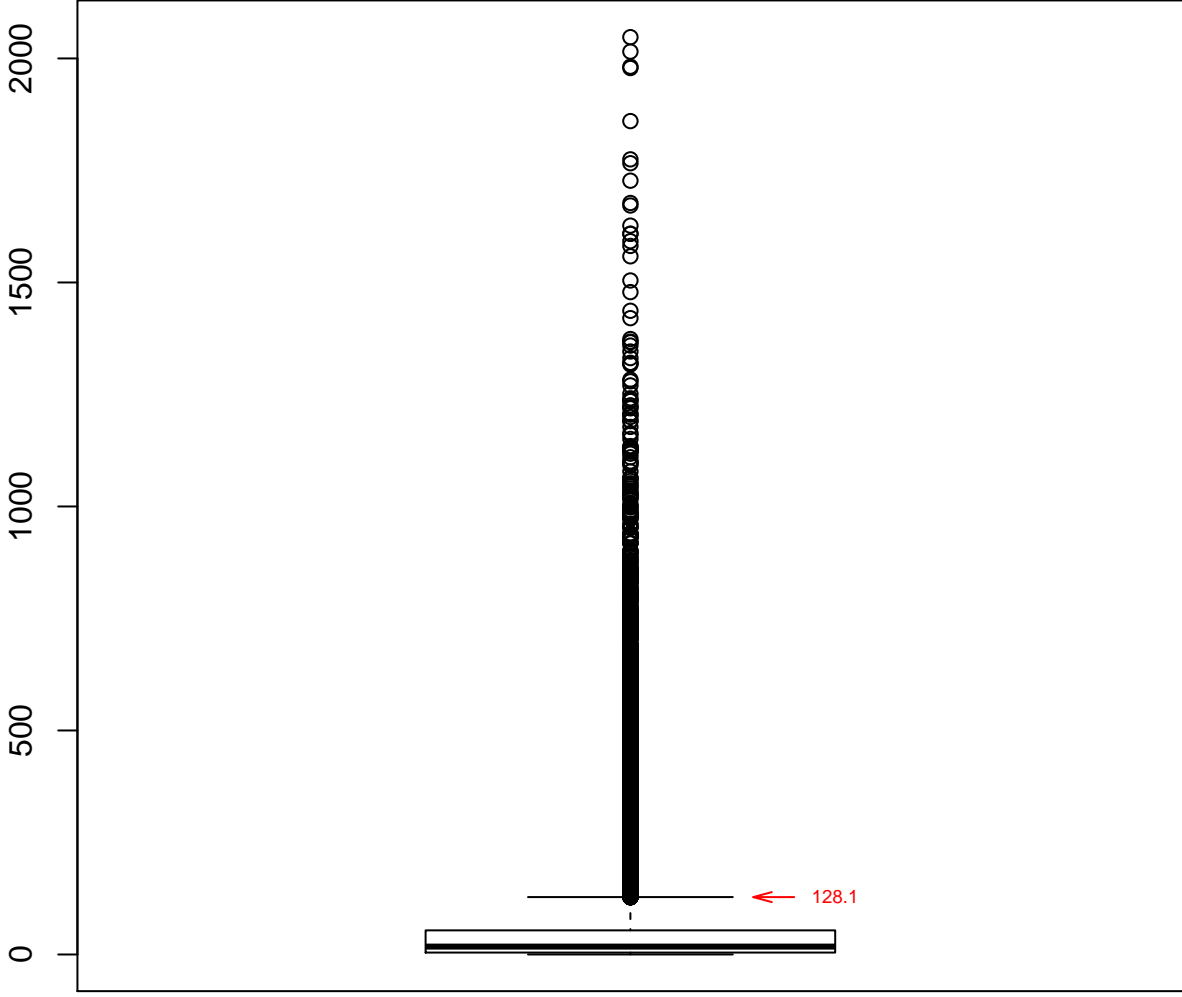

Supplement: S14 Fig — The BGV ranges from 0.051 to 2,047.575, but only 10.85% of the probesets show a BGV really high (greater than 128.1, the ‘maximum’ whisker). (PDF) [file pone.0157484.s016.pdf]

a)

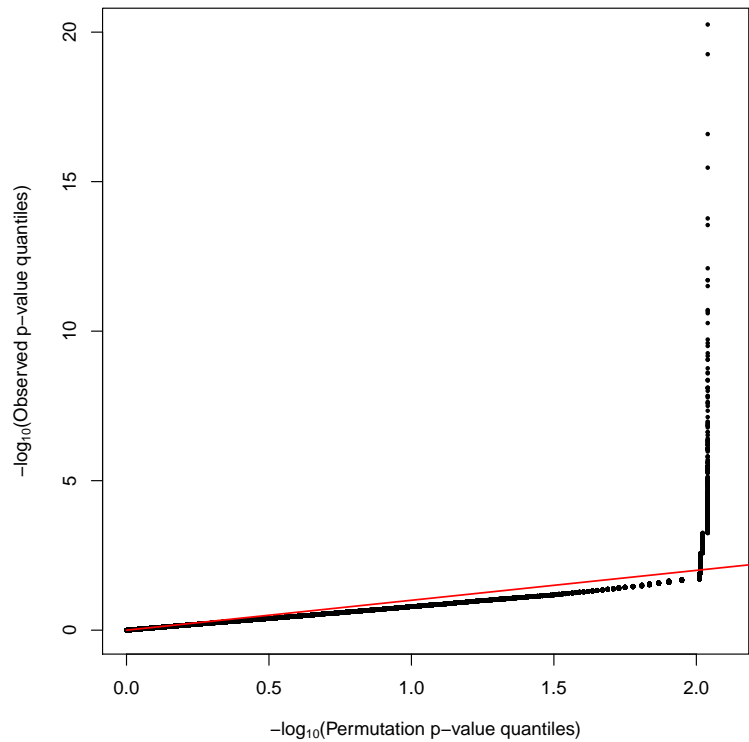

b)

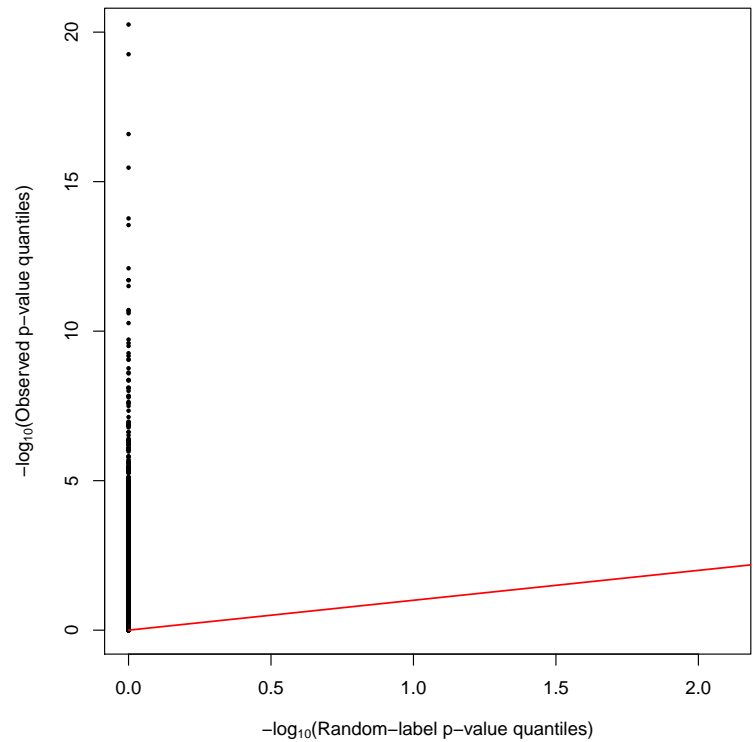

Supplement: S15 Fig — a) Permutation test QQ-plot. Quantiles of the adjusted permutation and observed p-values in log10 scale. Except for very extreme results observed due to resolution of attainable p-values in the permutation test, the observed p-values are larger than those obtained with the permutation test. b) QQ-plot of correct vs shuffled disease labels. After random permutation of the disease labels within each tissue type and multiple testing correction, none of the probesets are called significant. (PDF) [file pone.0157484.s017.pdf]

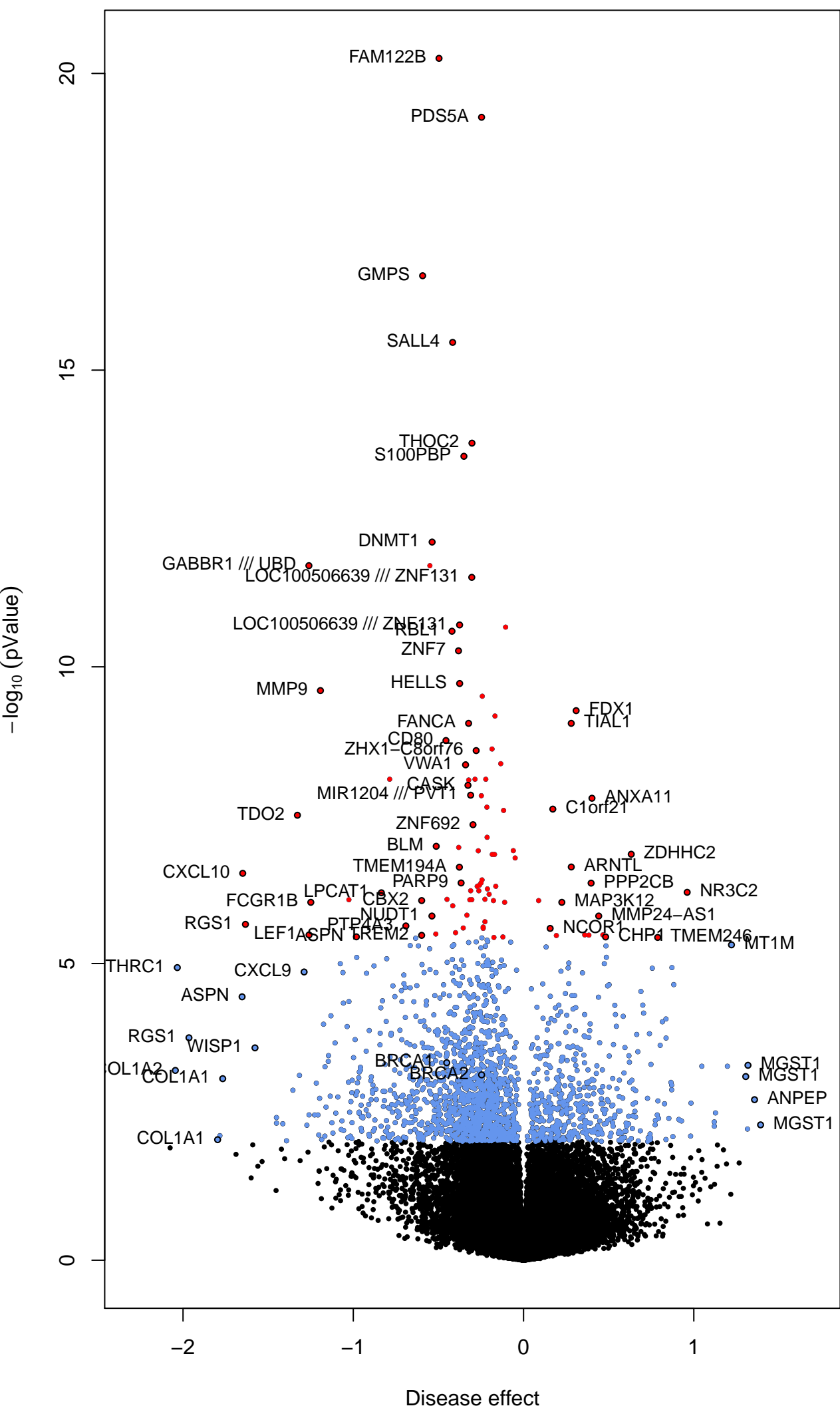

Supplement: S16 Fig — Plot of the disease effect, irrespective of the tissue type, versus the negative log 10-transformed p-values. (PDF) [file pone.0157484.s018.pdf]
