# Supplementary material for: Identification of Cancer Related Genes Using a Comprehensive Map of Human Gene Expression
Source: PLoS One. 2016 Jun 20;11(6):e0157484. doi: 10.1371/journal.pone.0157484 (PMC4913919; doi:10.1371/journal.pone.0157484)
Supplement: S9 Fig — Heatmap for the average pairwise correlations between samples from any two solid groups with at least 20 observations, accounting for the 10,000 most variable probesets in the computation of the correlations. The range for the similarity measure is (0.1704, 0.9896). The colour labels display smaller clusters in the hierarchical tree. (PDF) [file pone.0157484.s011.pdf]

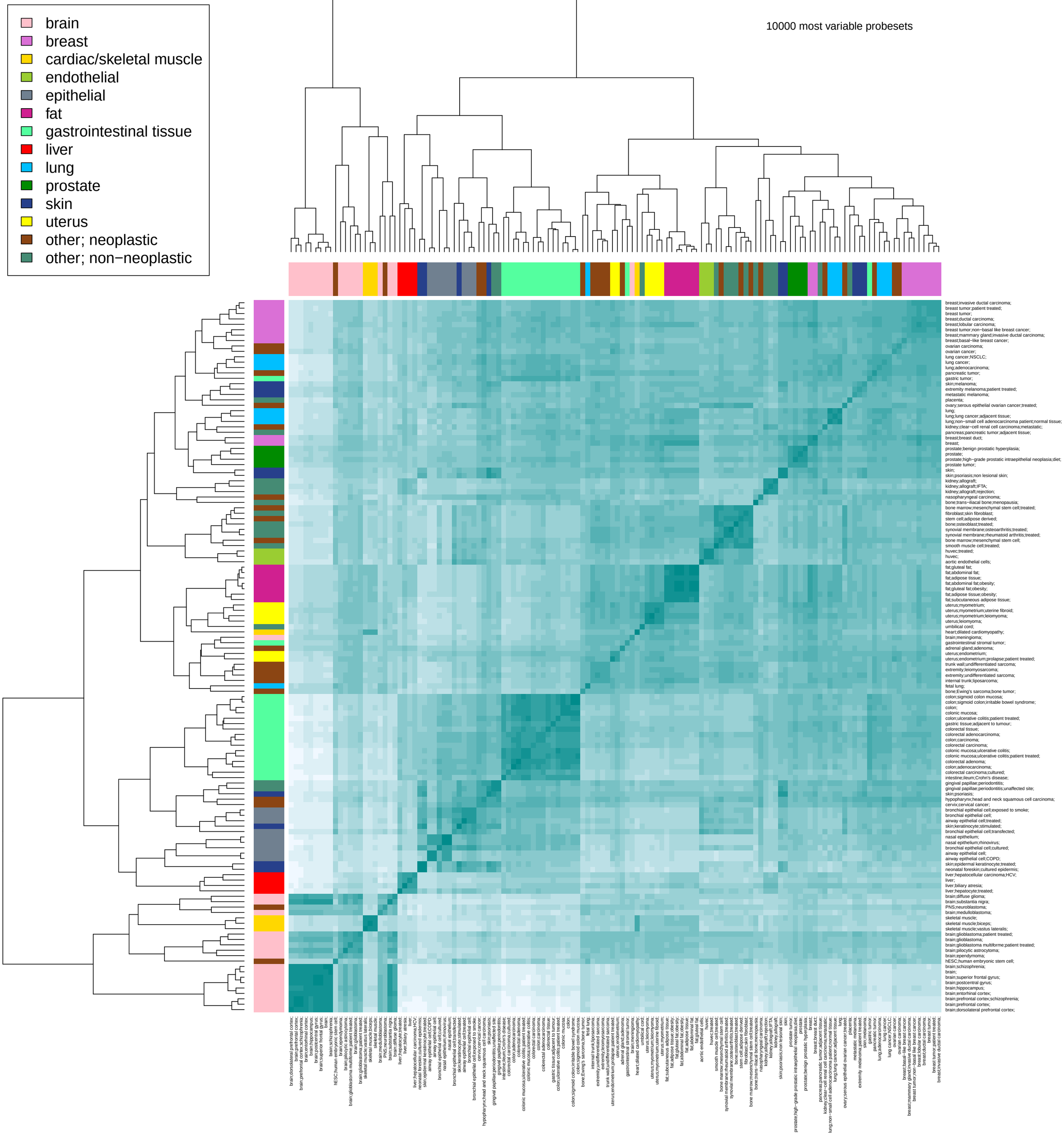
brain
breast
cardiac/skeletal muscle
endothelial
epithelial
fat
gastrointestinal tissue
liver
lung
prostate
skin
uterus
other; neoplastic
other; non−neoplastic
10000 most variable probesets
breast;invasive ductal carcinoma;
breast tumor;patient treated;
breast tumor;
breast;ductal carcinoma;
breast;lobular carcinoma;
breast tumor;non−basal like breast cancer;
breast;mammary gland;invasive ductal carcinoma;
breast;basal−like breast cancer;
ovarian carcinoma;
ovarian cancer;
lung cancer;NSCLC;
lung cancer;
lung;adenocarcinoma;
pancreatic tumor;
gastric tumor;
skin;melanoma;
extremity melanoma;patient treated;
metastatic melanoma;
placenta;
ovary;serous epithelial ovarian cancer;treated;
lung;
lung;lung cancer;adjacent tissue;
lung;non−small cell adenocarcinoma patient;normal tissue;
kidney;clear−cell renal cell carcinoma;metastatic;
pancreas;pancreatic tumor;adjacent tissue;
breast;breast duct;
breast;
prostate;benign prostatic hyperplasia;
prostate;
prostate;high−grade prostatic intraepithelial neoplasia;diet;
prostate tumor;
skin;
skin;psoriasis;non lesional skin;
kidney;allograft;
kidney;allograft;IFTA;
kidney;allograft;rejection;
nasopharyngeal carcinoma;
bone marrow;mesenchymal stem cell;treated;
bone;trans−iliacal bone;menopausia;
fibroblast;skin fibroblast;
stem cell;adipose derived;
bone;osteoblast;treated;
synovial membrane;osteoarthritis;treated;
synovial membrane;rheumatoid arthritis;treated;
bone marrow;mesenchymal stem cell;
smooth muscle cell;treated;
huvec;treated;
huvec;
aortic endothelial cells;
fat;gluteal fat;
fat;abdominal fat;
fat;adipose tissue;
fat;abdominal fat;obesity;
fat;gluteal fat;obesity;
fat;adipose tissue;obesity;
fat;subcutaneous adipose tissue;
uterus;myometrium;
uterus;myometrium;uterine fibroid;
uterus;myometrium;leiomyoma;
uterus;leiomyoma;
umbilical cord;
heart;dilated cardiomyopathy;
brain;meningioma;
gastrointestinal stromal tumor;
adrenal gland;adenoma;
uterus;endometrium;
uterus;endometrium;prolapse;patient treated;
trunk wall;undifferentiated sarcoma;
extremity;leiomyosarcoma;
extremity;undifferentiated sarcoma;
internal trunk;liposarcoma;
fetal lung;
bone;Ewing's sarcoma;bone tumor;
colon;sigmoid colon mucosa;
colon;sigmoid colon;irritable bowel syndrome;
colon;
colonic mucosa;
colon;ulcerative colitis;patient treated;
gastric tissue;adjacent to tumour;
colorectal tissue;
colorectal adenocarcinoma;
colon;carcinoma;
colorectal carcinoma;
colonic mucosa;ulcerative colitis;
colonic mucosa;ulcerative colitis;patient treated;
colorectal adenoma;
colon;adenocarcinoma;
colorectal carcinoma;cultured;
intestine;ileum;Crohn's disease;
gingival papillae;periodontitis;
gingival papillae;periodontitis;unaffected site;
skin;psoriasis;
hypopharynx;head and neck squamous cell carcinoma;
cervix;cervical cancer;
bronchial epithelial cell;exposed to smoke;
bronchial epithelial cell;
airway epithelial cell;treated;
skin;keratinocyte;stimulated;
bronchial epithelial cell;transfected;
nasal epithelium;
nasal epithelium;rhinovirus;
bronchial epithelial cell;cultured;
airway epithelial cell;
airway epithelial cell;COPD;
skin;epidermal keratinocyte;treated;
neonatal foreskin;cultured epidermis;
liver;hepatocellular carcinoma;HCV;
liver;
liver;biliary atresia;
liver;hepatocyte;treated;
brain;diffuse glioma;
brain;substantia nigra;
PNS;neuroblastoma;
brain;medulloblastoma;
skeletal muscle;
skeletal muscle;biceps;
skeletal muscle;vastus lateralis;
brain;glioblastoma;patient treated;
brain;glioblastoma;
brain;glioblastoma multiforme;patient treated;
brain;pilocytic astrocytoma;
brain;ependymoma;
hESC;human embryonic stem cell;
brain;schizophrenia;
brain;
brain;superior frontal gyrus;
brain;postcentral gyrus;
brain;hippocampus;
brain;entorhinal cortex;
brain;prefrontal cortex;schizophrenia;
brain;prefrontal cortex;
brain;dorsolateral prefrontal cortex;
