# Supplementary material for: Identification of Cancer Related Genes Using a Comprehensive Map of Human Gene Expression
Source: PLoS One. 2016 Jun 20;11(6):e0157484. doi: 10.1371/journal.pone.0157484 (PMC4913919; doi:10.1371/journal.pone.0157484)
Supplement: S10 Fig — Heatmap for the average pairwise correlations between samples from any two solid groups with at least 20 observations, accounting for the 5,000 most variable probesets in the computation of the correlations. The range for the similarity measure is (0.0247, 0.9893). The colour labels display smaller clusters in the hierarchical tree. (PDF) [file pone.0157484.s012.pdf]

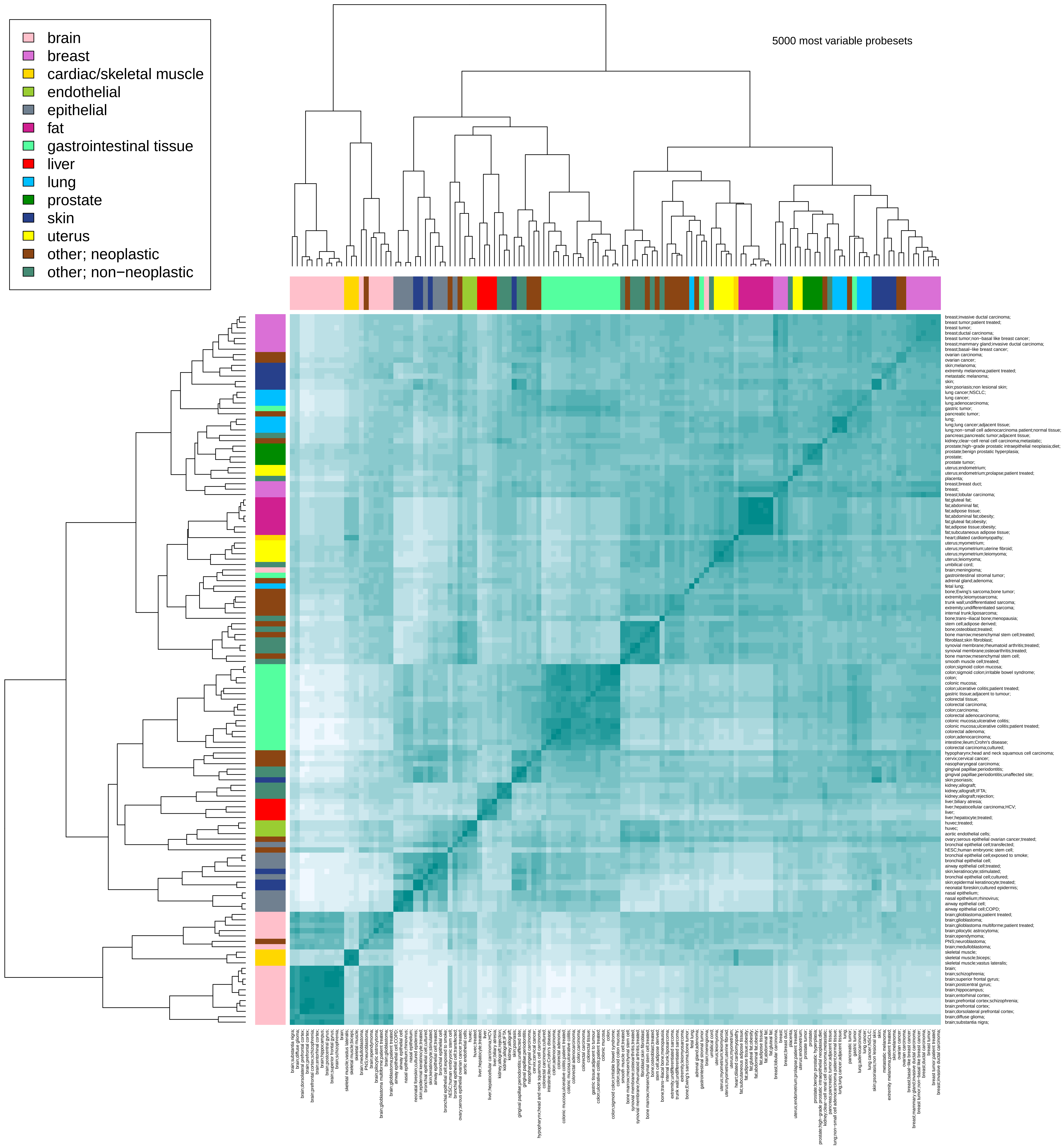
brain
breast
cardiac/skeletal muscle
endothelial
epithelial
fat
gastrointestinal tissue
liver
lung
prostate
skin
uterus
other; neoplastic
other; non−neoplastic
5000 most variable probesets
breast;invasive ductal carcinoma;
breast tumor;patient treated;
breast tumor;
breast;ductal carcinoma;
breast tumor;non−basal like breast cancer;
breast;mammary gland;invasive ductal carcinoma;
breast;basal−like breast cancer;
ovarian carcinoma;
ovarian cancer;
skin;melanoma;
extremity melanoma;patient treated;
metastatic melanoma;
skin;
skin;psoriasis;non lesional skin;
lung cancer;NSCLC;
lung cancer;
lung;adenocarcinoma;
gastric tumor;
pancreatic tumor;
lung;
lung;lung cancer;adjacent tissue;
lung;non−small cell adenocarcinoma patient;normal tissue;
pancreas;pancreatic tumor;adjacent tissue;
kidney;clear−cell renal cell carcinoma;metastatic;
prostate;high−grade prostatic intraepithelial neoplasia;diet;
prostate;benign prostatic hyperplasia;
prostate;
prostate tumor;
uterus;endometrium;
uterus;endometrium;prolapse;patient treated;
placenta;
breast;breast duct;
breast;
breast;lobular carcinoma;
fat;gluteal fat;
fat;abdominal fat;
fat;adipose tissue;
fat;abdominal fat;obesity;
fat;gluteal fat;obesity;
fat;adipose tissue;obesity;
fat;subcutaneous adipose tissue;
heart;dilated cardiomyopathy;
uterus;myometrium;
uterus;myometrium;uterine fibroid;
uterus;myometrium;leiomyoma;
uterus;leiomyoma;
umbilical cord;
brain;meningioma;
gastrointestinal stromal tumor;
adrenal gland;adenoma;
fetal lung;
bone;Ewing's sarcoma;bone tumor;
extremity;leiomyosarcoma;
trunk wall;undifferentiated sarcoma;
extremity;undifferentiated sarcoma;
internal trunk;liposarcoma;
bone;trans−iliacal bone;menopausia;
stem cell;adipose derived;
bone;osteoblast;treated;
bone marrow;mesenchymal stem cell;treated;
fibroblast;skin fibroblast;
synovial membrane;rheumatoid arthritis;treated;
synovial membrane;osteoarthritis;treated;
bone marrow;mesenchymal stem cell;
smooth muscle cell;treated;
colon;sigmoid colon mucosa;
colon;sigmoid colon;irritable bowel syndrome;
colon;
colonic mucosa;
colon;ulcerative colitis;patient treated;
gastric tissue;adjacent to tumour;
colorectal tissue;
colorectal carcinoma;
colon;carcinoma;
colorectal adenocarcinoma;
colonic mucosa;ulcerative colitis;
colonic mucosa;ulcerative colitis;patient treated;
colorectal adenoma;
colon;adenocarcinoma;
intestine;ileum;Crohn's disease;
colorectal carcinoma;cultured;
hypopharynx;head and neck squamous cell carcinoma;
cervix;cervical cancer;
nasopharyngeal carcinoma;
gingival papillae;periodontitis;
gingival papillae;periodontitis;unaffected site;
skin;psoriasis;
kidney;allograft;
kidney;allograft;IFTA;
kidney;allograft;rejection;
liver;biliary atresia;
liver;hepatocellular carcinoma;HCV;
liver;
liver;hepatocyte;treated;
huvec;treated;
huvec;
aortic endothelial cells;
ovary;serous epithelial ovarian cancer;treated;
bronchial epithelial cell;transfected;
hESC;human embryonic stem cell;
bronchial epithelial cell;exposed to smoke;
bronchial epithelial cell;
airway epithelial cell;treated;
skin;keratinocyte;stimulated;
bronchial epithelial cell;cultured;
skin;epidermal keratinocyte;treated;
neonatal foreskin;cultured epidermis;
nasal epithelium;
nasal epithelium;rhinovirus;
airway epithelial cell;
airway epithelial cell;COPD;
brain;glioblastoma;patient treated;
brain;glioblastoma;
brain;glioblastoma multiforme;patient treated;
brain;pilocytic astrocytoma;
brain;ependymoma;
PNS;neuroblastoma;
brain;medulloblastoma;
skeletal muscle;
skeletal muscle;biceps;
skeletal muscle;vastus lateralis;
brain;
brain;schizophrenia;
brain;superior frontal gyrus;
brain;postcentral gyrus;
brain;hippocampus;
brain;entorhinal cortex;
brain;prefrontal cortex;schizophrenia;
brain;prefrontal cortex;
brain;dorsolateral prefrontal cortex;
brain;diffuse glioma;
brain;substantia nigra;
