# Supplementary material for: Identification of Cancer Related Genes Using a Comprehensive Map of Human Gene Expression
Source: PLoS One. 2016 Jun 20;11(6):e0157484. doi: 10.1371/journal.pone.0157484 (PMC4913919; doi:10.1371/journal.pone.0157484)
Supplement: S11 Fig — Heatmap for the average pairwise correlations between samples from any two solid groups with at least 20 observations, accounting for the 1,000 most variable probesets in the computation of the correlations. The range for the similarity measure is (−0.1312, 0.9938). The colour labels display smaller clusters in the hierarchical tree. (PDF) [file pone.0157484.s013.pdf]

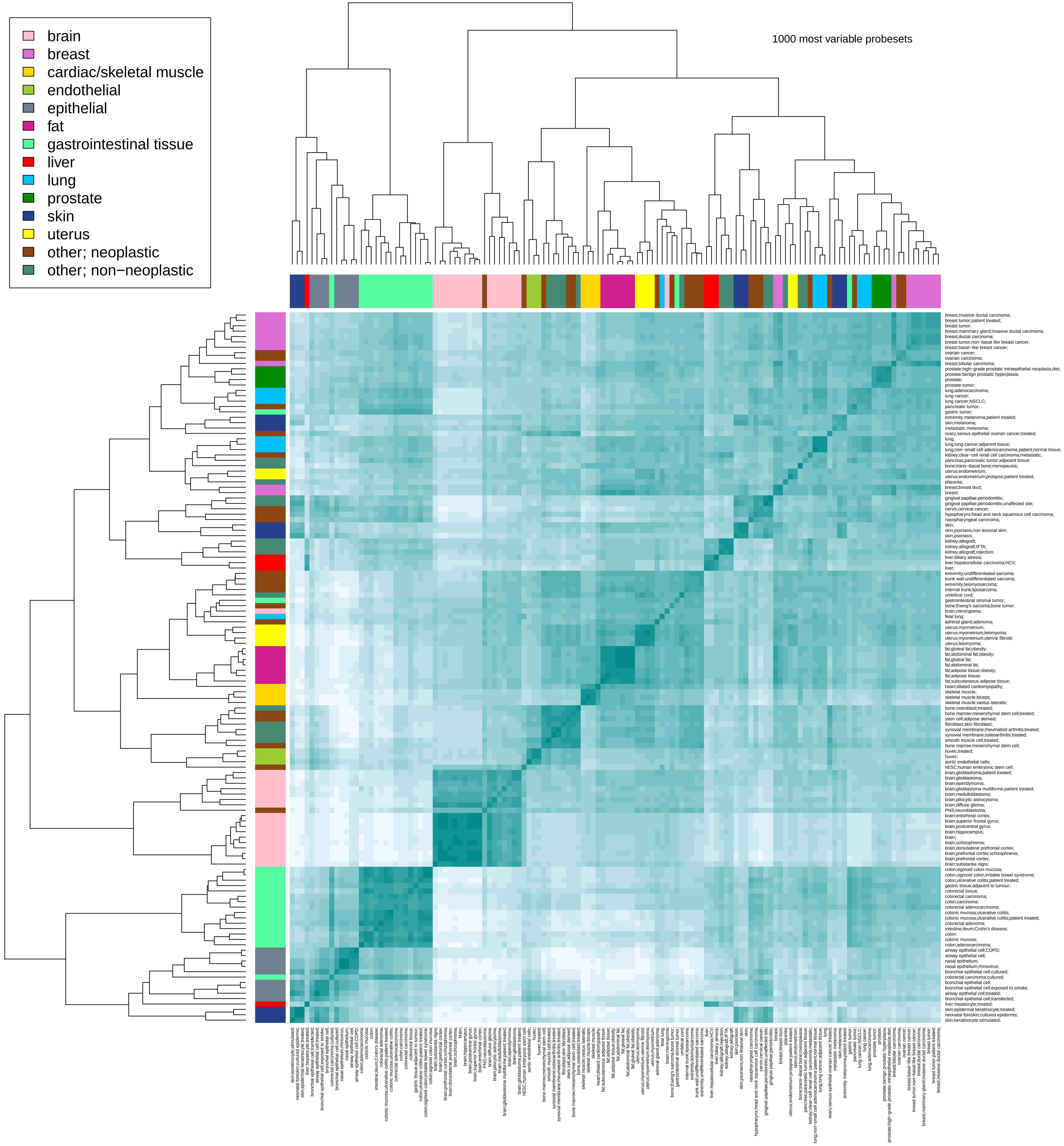
brain
breast
cardiac/skeletal muscle
endothelial
epithelial
fat
gastrointestinal tissue
liver
lung
prostate
skin
uterus
other; neoplastic
other; non−neoplastic
1000 most variable probesets
breast;invasive ductal carcinoma;
breast tumor;patient treated;
breast tumor;
breast;mammary gland;invasive ductal carcinoma;
breast;ductal carcinoma;
breast tumor;non−basal like breast cancer;
breast;basal−like breast cancer;
ovarian cancer;
ovarian carcinoma;
breast;lobular carcinoma;
prostate;high−grade prostatic intraepithelial neoplasia;diet;
prostate;benign prostatic hyperplasia;
prostate;
prostate tumor;
lung;adenocarcinoma;
lung cancer;
lung cancer;NSCLC;
pancreatic tumor;
gastric tumor;
extremity melanoma;patient treated;
skin;melanoma;
metastatic melanoma;
ovary;serous epithelial ovarian cancer;treated;
lung;
lung;lung cancer;adjacent tissue;
lung;non−small cell adenocarcinoma patient;normal tissue;
kidney;clear−cell renal cell carcinoma;metastatic;
pancreas;pancreatic tumor;adjacent tissue;
bone;trans−iliacal bone;menopausia;
uterus;endometrium;
uterus;endometrium;prolapse;patient treated;
placenta;
breast;breast duct;
breast;
gingival papillae;periodontitis;
gingival papillae;periodontitis;unaffected site;
cervix;cervical cancer;
hypopharynx;head and neck squamous cell carcinoma;
nasopharyngeal carcinoma;
skin;
skin;psoriasis;non lesional skin;
skin;psoriasis;
kidney;allograft;
kidney;allograft;IFTA;
kidney;allograft;rejection;
liver;biliary atresia;
liver;hepatocellular carcinoma;HCV;
liver;
extremity;undifferentiated sarcoma;
trunk wall;undifferentiated sarcoma;
extremity;leiomyosarcoma;
internal trunk;liposarcoma;
umbilical cord;
gastrointestinal stromal tumor;
bone;Ewing's sarcoma;bone tumor;
brain;meningioma;
fetal lung;
adrenal gland;adenoma;
uterus;myometrium;
uterus;myometrium;leiomyoma;
uterus;myometrium;uterine fibroid;
uterus;leiomyoma;
fat;gluteal fat;obesity;
fat;abdominal fat;obesity;
fat;gluteal fat;
fat;abdominal fat;
fat;adipose tissue;obesity;
fat;adipose tissue;
fat;subcutaneous adipose tissue;
heart;dilated cardiomyopathy;
skeletal muscle;
skeletal muscle;biceps;
skeletal muscle;vastus lateralis;
bone;osteoblast;treated;
bone marrow;mesenchymal stem cell;treated;
stem cell;adipose derived;
fibroblast;skin fibroblast;
synovial membrane;rheumatoid arthritis;treated;
synovial membrane;osteoarthritis;treated;
smooth muscle cell;treated;
bone marrow;mesenchymal stem cell;
huvec;treated;
huvec;
aortic endothelial cells;
hESC;human embryonic stem cell;
brain;glioblastoma;patient treated;
brain;glioblastoma;
brain;ependymoma;
brain;glioblastoma multiforme;patient treated;
brain;medulloblastoma;
brain;pilocytic astrocytoma;
brain;diffuse glioma;
PNS;neuroblastoma;
brain;entorhinal cortex;
brain;superior frontal gyrus;
brain;postcentral gyrus;
brain;hippocampus;
brain;
brain;schizophrenia;
brain;dorsolateral prefrontal cortex;
brain;prefrontal cortex;schizophrenia;
brain;prefrontal cortex;
brain;substantia nigra;
colon;sigmoid colon mucosa;
colon;sigmoid colon;irritable bowel syndrome;
colon;ulcerative colitis;patient treated;
gastric tissue;adjacent to tumour;
colorectal tissue;
colorectal carcinoma;
colon;carcinoma;
colorectal adenocarcinoma;
colonic mucosa;ulcerative colitis;
colonic mucosa;ulcerative colitis;patient treated;
colorectal adenoma;
intestine;ileum;Crohn's disease;
colon;
colonic mucosa;
colon;adenocarcinoma;
airway epithelial cell;COPD;
airway epithelial cell;
nasal epithelium;
nasal epithelium;rhinovirus;
bronchial epithelial cell;cultured;
colorectal carcinoma;cultured;
bronchial epithelial cell;
bronchial epithelial cell;exposed to smoke;
airway epithelial cell;treated;
bronchial epithelial cell;transfected;
liver;hepatocyte;treated;
skin;epidermal keratinocyte;treated;
neonatal foreskin;cultured epidermis;
skin;keratinocyte;stimulated;
