# Supplementary material for: Identification of Cancer Related Genes Using a Comprehensive Map of Human Gene Expression
Source: PLoS One. 2016 Jun 20;11(6):e0157484. doi: 10.1371/journal.pone.0157484 (PMC4913919; doi:10.1371/journal.pone.0157484)
Supplement: S12 Fig — Heatmap for the average pairwise correlations between samples from any two solid groups with at least 20 observations, accounting for the 500 most variable probesets in the computation of the correlations. The range for the similarity measure is (−0.2424, 0.9955). The colour labels display smaller clusters in the hierarchical tree. (PDF) [file pone.0157484.s014.pdf]

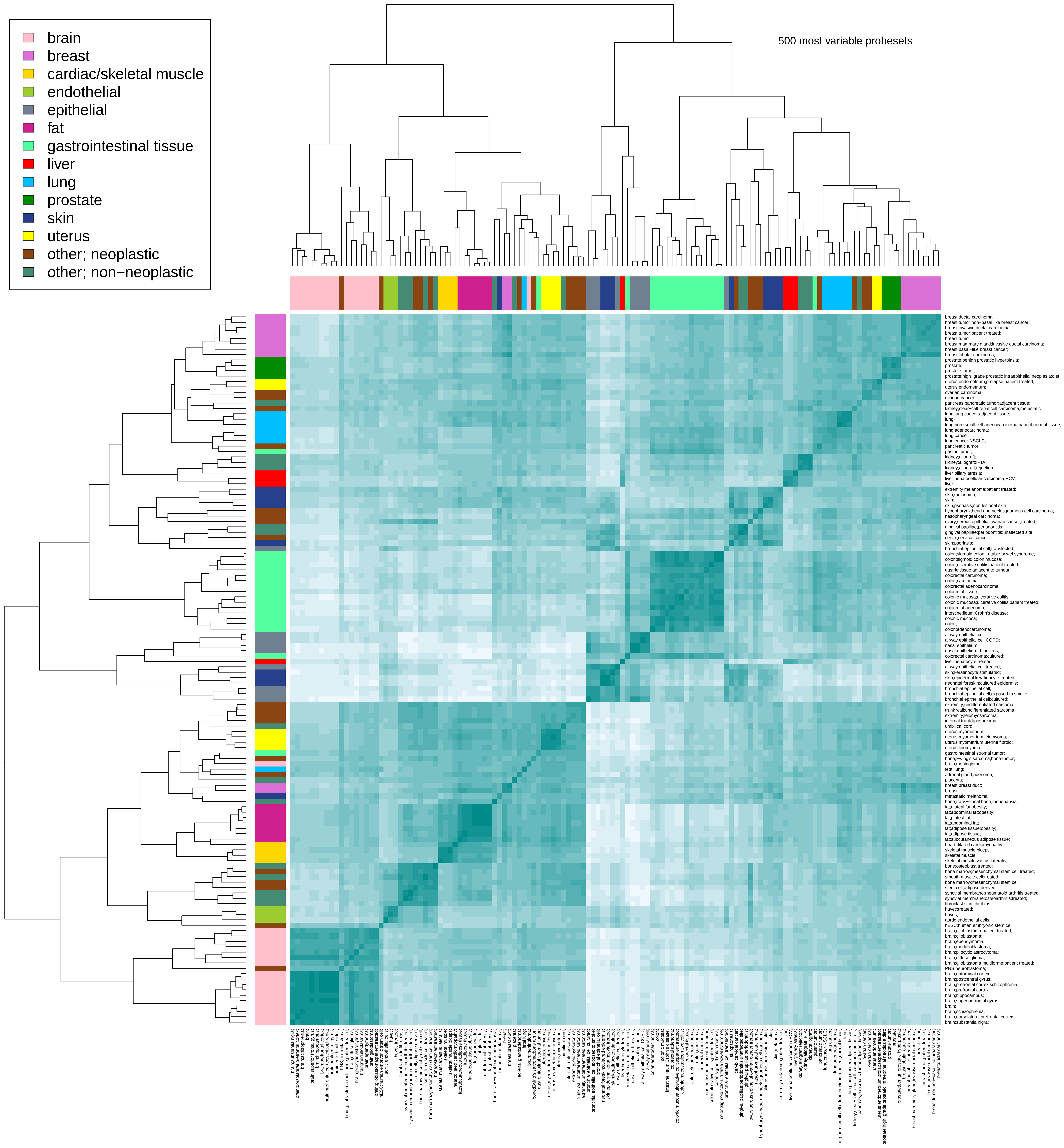
500 most variable probesets
brain
breast
cardiac/skeletal muscle
endothelial
epithelial
fat
gastrointestinal tissue
liver
lung
prostate
skin
uterus
other; neoplastic
other; non−neoplastic
