# Supplementary material for: Identification of Cancer Related Genes Using a Comprehensive Map of Human Gene Expression
Source: PLoS One. 2016 Jun 20;11(6):e0157484. doi: 10.1371/journal.pone.0157484 (PMC4913919; doi:10.1371/journal.pone.0157484)
Supplement: S13 Fig — Heatmap for the expression level of the 1,000 most variable probesets averaged over the samples included in each biological group with at least 20 observations. The range for this similarity measure is (2.6984, 14.4581). The colour labels display the same clusters as those in S11 Fig. The probeset labels report the name of the genes they are mapping to. Probesets mapping to the same gene are clustered together. (PDF) [file pone.0157484.s015.pdf]

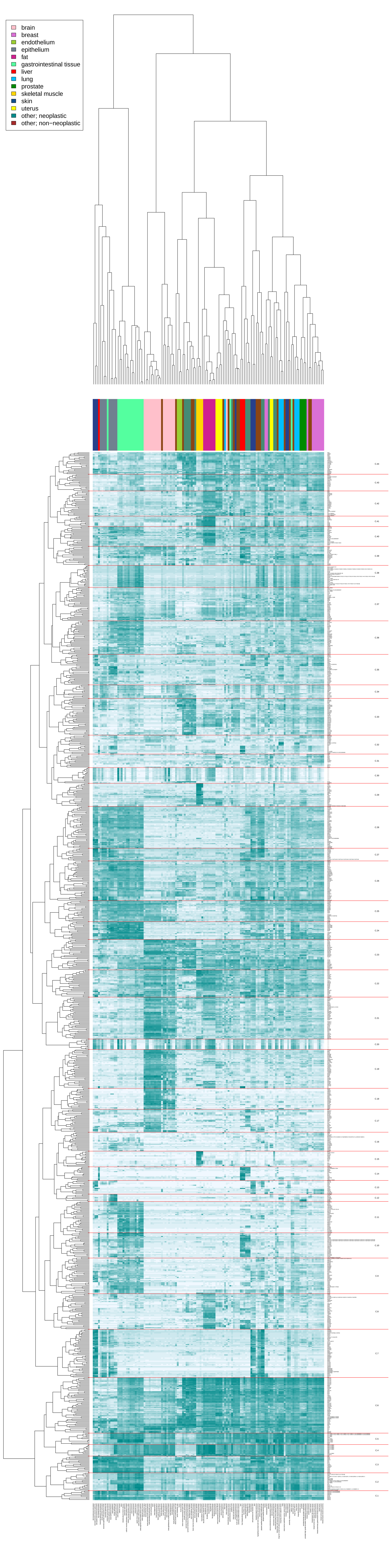
brain
breast
endothelium
epithelium
fat
gastrointestinal tissue
liver
lung
prostate
skeletal muscle
skin
uterus
other; neoplastic
other; non−neoplastic
